# Silver phosphate-modified carbonate apatite honeycomb scaffolds for anti-infective and pigmentation-free bone tissue engineering

**DOI:** 10.1016/j.mtbio.2024.101161

**Published:** 2024-07-18

**Authors:** Koichiro Hayashi, Masaya Shimabukuro, Cheng Zhang, Ahmad Nazir Taleb Alashkar, Ryo Kishida, Akira Tsuchiya, Kunio Ishikawa

**Affiliations:** a Department of Biomaterials, Faculty of Dental Science, Kyushu University, 3-1-1 Maidashi, Higashi-ku, Fukuoka, 812-8582, Japan; b Institute of Biomaterials and Bioengineering, Tokyo Medical and Dental University, 2-3-10, Kanda-Surugadai, Chiyoda-ku, Tokyo, 101-0062, Japan

**Keywords:** Scaffolds, Anti-infection, Bone tissue engineering, Honeycomb, Apatite

## Abstract

Bone regeneration using synthetic materials has a high rate of surgical site infection, resulting in severe pain for patients and often requiring revision surgery. We propose Ag_3_PO_4_-based surface modification and structural control of scaffolds for preventing infections in bone regeneration. We demonstrated the differences in toxicity and antibacterial activity between in vitro and in vivo studies and determined the optimal silver content in terms of overall anti-infection effects, bone regeneration, toxicity, and pigmentation. A honeycomb structure comprising osteoconductive and resorbable carbonate apatite (CAp) was used as the base scaffold. CAp in the scaffold surface was partially replaced with different concentrations of Ag_3_PO_4_ via controlled dissolution-precipitation reactions in an AgNO_3_ solution. Both bone regeneration and infection prevention were achieved at 860–2300 ppm of silver. Despite the absence of Ag_3_PO_4_, honeycomb scaffolds were less susceptible to infection, even under conditions where infection occurs in clinically used three-dimensional porous scaffolds. Regardless of in vitro cytotoxicity at >5200 ppm of silver, increasing the silver content to 21,000 ppm did not adversely affect in vivo bone formation and scaffold resorption or cause acute systemic toxicity. Rather, bone formation was enhanced with 5200 ppm of silver. However, pigmentation was observed at that concentration. Hence, we concluded that the optimal silver concentration range is 860–2300 ppm for anti-infective and pigmentation-free bone regeneration. Bone regeneration was achieved via surface modification, resulting in the rapid release of silver ions immediately after implantation, followed by gradual release over several months. The scaffold structure may also aid in preventing bacterial growth within the scaffolds.

## Graphical abstract

**Figure:**
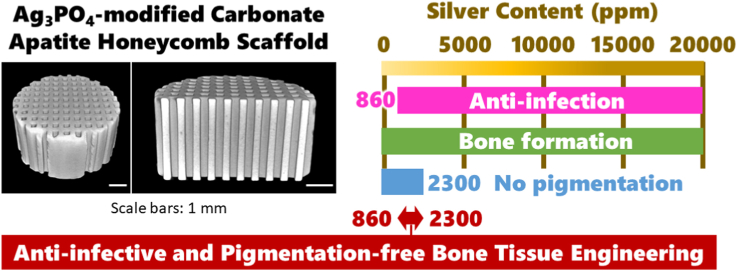

## 1 Introduction

Maintaining the functions of bone, which is part of the locomotor system, significantly improves the quality of life and extends healthy life expectancy [1]. Autologous bone grafts, allografts, and xenografts are conventionally used for repairing bone defects [1,2]. However, autologous bone grafts are less frequently harvested as the process is invasive, and allografts and xenografts are immunogenic. To resolve the difficulties of these conventional grafts, synthetic scaffolds for bone tissue engineering have been developed [2,3]. However, synthetic scaffolds often cause infections because bacteria can easily adhere to and grow on them [[4], [5], [6], [7], [8], [9], [10], [11], [12], [13]]. Furthermore, biofilms form on the surfaces of infected synthetic scaffolds, and antibacterial drugs become less effective [[8], [9], [10], [11], [12], [13]]. Consequently, several patients have required repeated surgeries.

Researchers have attempted to prevent bacterial infections by combining synthetic scaffolds with antibacterial agents [[4], [5], [6], [7]]. Among the various organic and inorganic antibacterial agents, silver agents have exhibited durability against sterilization and a broad antibacterial spectrum while also producing less drug-resistant bacteria [[4], [5], [6], [7],[14], [15], [16], [17], [18], [19]]. Silver ions show stronger antibacterial activity than other metal ions, such as copper, zinc, cobalt, and gallium [4,5,14,20]. Moreover, previous in vitro studies have demonstrated that silver ions enhance the proliferation and differentiation of mesenchymal stem cells and osteoblast-like cells [[21], [22], [23], [24], [25]]. Therefore, a combination of synthetic scaffolds and silver compounds is considered a rational approach.

In this context, composites of synthetic osteoconductive scaffolds and silver compounds have been developed [[26], [27], [28], [29], [30], [31], [32], [33], [34], [35], [36], [37], [38], [39], [40], [41], [42], [43], [44]]. The primary combination approaches include doping silver ions into calcium phosphate crystals, dispersing silver nanoparticles and compounds into the scaffold, and coating the scaffold with silver compounds [4,17]. However, to prevent surgical site infections and reduce the health risks caused by long-term exposure to silver ions, such as argyrosis, a modification that provides a higher concentration of silver ions on the outer surface and a lower concentration of silver ions in the interior of the scaffold seems reasonable.

Furthermore, the biodegradable properties of the scaffold are critical for bone regeneration while preventing infections [45]. For infection prevention, scaffolds should be bioresorbable because the cause of infection is eliminated once the scaffold is resorbed [45]. However, little bone is formed when scaffold resorption is faster than bone formation [45]. Therefore, the scaffolds should be gradually resorbed after bone formation. In terms of biodegradability, carbonate apatite (CAp), which is a bone mineral analog, is suitable for scaffold composition because it does not spontaneously dissolve and is gradually resorbed by the weak acids produced by osteoclasts [[46], [47], [48], [49]].

The scaffold structure should also be adequately considered because it affects bone regeneration and infection prevention [[50], [51], [52], [53], [54], [55]]. To achieve rapid bone regeneration, scaffolds must possess structures that promote the ingrowth of bone and blood vessels. We have demonstrated that a honeycomb (HC) structure is superior to two- and three-dimensional (3D) porous structures in the formation of bone and blood vessels [[56], [57], [58], [59], [60]]. Furthermore, the scaffold structure may affect the ease of bacterial attachment to the scaffold and susceptibility to infection. Our previous study suggested that HC scaffolds are less susceptible to infection because this structure facilitates blood inflow and prevents bacterial adhesion to the scaffold [40]. However, the aforementioned study did not compare the anti-infective activities of HC and other structures. Hence, a comparison between HC and poorly interconnected porous structures is necessary to elucidate the structural effects on anti-infective activity.

Most previous studies on silver-containing osteoconductive ceramic scaffolds have only performed in vitro antibacterial and cytotoxic evaluations [[27], [28], [29], [30], [31], [32], [33], [34], [35], [36], [37], [38], [39],[41], [42], [43], [44],[61], [62], [63]], and only a few studies have investigated in vivo infection prevention and bone regeneration [40,64]. Furthermore, although the osteogenesis-promoting effects of silver ions have been demonstrated in vitro, the promotion of bone formation by silver ions in vivo remains unclear. Moreover, although silver ions react with sulfur in vivo to form silver sulfide and cause pigmentation, the concentration of silver at which pigmentation begins is unknown. Thus, from a comprehensive perspective of anti-infection, bone regeneration, toxicity, and pigmentation, the optimal range of silver concentrations should be clarified through in vivo evaluations.

In this study, we aimed to clarify the optimal silver concentration range for achieving anti-infective and pigmentation-free bone tissue engineering by in vivo evaluations using CAp HC scaffolds modified with various concentrations of Ag_3_PO_4_. Furthermore, we aimed to determine the effects of the scaffold structure on infection prevention and bone regeneration by comparing HC scaffolds with clinically used 3D porous scaffolds.

## 2 Materials and methods

### 2.1 Fabrication of CAp HC scaffolds

The CAp HC scaffolds were fabricated based on the procedures developed in our previous study [65]. First, a mixture of CaCO_3_ powder (Sakai Chemical, Osaka, Japan) and a methylcellulose-based binder (Universe, Saga, Japan) was prepared using a mixer (D-10, Universe, Saga, Japan). Second, HC sticks of the mixture were extruded through a die—comprising a square lattice and slit width of 300 μm—which was fixed to an extruder (V-30 (II), Universe). Third, the HC sticks were cut into 5 mm-thick sticks, and the binder was removed by heating at 520 °C for 96 h under air and then at 650 °C for 24 h under the flow of CO_2_. After these procedures, CaCO_3_ HC structures were obtained. Finally, the CaCO_3_ HC structures were immersed in 1 mol/L Na_2_HPO_4_ aqueous solution at 80 °C for seven days, which converted the composition from CaCO_3_ to CAp. The obtained CAp HC scaffolds were then shaped into cylinders with a diameter of 6 mm and height of 4 mm using a 3D milling machine (monoFab SRM-20, Roland DG, Shizuoka, Japan).

### 2.2 Surface modification of CAp HC scaffolds with Ag_3_PO_4_

**Fig. 1:**
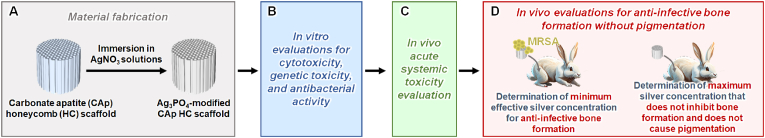
Experimental scheme. (**A**) Surface modification of carbonate apatite (CAp) honeycomb (HC) scaffolds with Ag_3_PO_4_. (**B**) In vitro evaluations for cytotoxicity, genetic toxicity, and antibacterial activity. (**C**) In vivo acute systemic toxicity evaluation to clarify the gap between in vitro and in vivo toxicities and determine the Ag_3_PO_4_-modified CAp HC scaffold with maximum silver content permissible for use in vivo bone formation evaluations. (**D**) In vivo evaluations to determine the minimum effective silver concentration for anti-infective bone formation and the maximum silver concentration that neither inhibits bone formation nor causes pigmentation.

Ag_3_PO_4_-modified CAp HC scaffolds were prepared by immersing CAp HC scaffolds in AgNO_3_ aqueous solutions (0.05, 0.1, 0.3, 0.6, 1, 2, 5, 10, 15, or 20 mmol/L) at 20 °C for 1 h to partially replace CAp in the surface regions of HC scaffolds with Ag_3_PO_4_ (Fig. 1A). The Ag_3_PO_4_-modified CAp HC scaffolds were washed five times with distilled water and stored in a dark environment. The different concentrations of AgNO_3_, corresponding silver contents in the scaffolds, and their designated names are listed in Table 1.

**Table 1:** **Conditions for Ag**_**3**_**PO**_**4**_**modification of carbonate apatite honeycomb (CAp HC) scaffolds and designated sample names.**^a^Silver contents were measured via inductively coupled plasma atomic emission spectrometry. ^b^Sample names were designated based on the values that were rounded off to one place of the average measured silver contents in the HC scaffolds.

AgNO_3_ solution (mmol/L)	Silver content (ppm)^a^	Designated scaffold name^b^
0	0	Ag0-HC
0.05	40 ± 7	Ag40-HC
0.1	110 ± 7	Ag110-HC
0.3	183 ± 5	Ag180-HC
0.6	285 ± 11	Ag290-HC
1	475 ± 15	Ag480-HC
2	864 ± 179	Ag860-HC
5	2300 ± 150	Ag2300-HC
10	5200 ± 471	Ag5200-HC
15	13,667 ± 471	Ag13670-HC
20	21,000 ± 433	Ag21000-HC

### 2.3 Characterization of physicochemical, structural, and mechanical properties

The crystal phases and functional groups of Ag_3_PO_4_-modified CAp HC scaffolds were identified via X-ray diffraction (XRD) using CuKα radiation at 40 kV and 40 mA (Rigaku, D8 Advance, Bruker, MA, USA) and Fourier transform infrared (FTIR) spectroscopy using the KBr tablet method (FT/IR-6200, JASCO, Tokyo, Japan), respectively. Commercial Ag_3_PO_4_ powder (Fujifilm Wako Chemical, Osaka, Japan) was used as the reference material. X-ray photoelectron spectroscopy (XPS) profiles of CAp HC scaffolds before and after Ag_3_PO_4_ modification were obtained using a K-alpha spectrophotometer (Thermo Fisher Scientific, East Grinstead, UK) with an Al Kα X-ray source (energy: 1486.6 eV). Carbon and nitrogen in CAp HC scaffolds before and after Ag_3_PO_4_ modification were quantified using a CHN elemental analyzer (MT-6, Yanaco, Kyoto, Japan).

The overall and internal structures of CAp HC scaffolds before and after Ag_3_PO_4_ modification and those of the clinically used 3D porous scaffold (Osferion60-Marvelous, Olympus Terumo Biomaterials, Tokyo, Japan) were observed via micro-computed tomography (μ-CT; ScanXmate-L090T, Comscan, Kanagawa, Japan). Microstructural observations and surface elemental analysis of the Ag_3_PO_4_-modified CAp HC scaffolds were conducted using scanning electron microscopy–energy dispersive X-ray (SEM–EDX; S–3400 N, Hitachi High Technology, Tokyo, Japan).

The silver content of the Ag_3_PO_4_-modified CAp HC scaffolds was quantified by completely dissolving the scaffolds in a 2 % HNO_3_ aqueous solution; the obtained solutions were injected into an inductively coupled plasma atomic emission spectrometer (ICP-AES; Optima 7300 DV, PerkinElmer, MA, USA). Silver standard solution (Fujifilm Wako Chemical) was used to prepare the calibration curve. Similarly, after storing the Ag_3_PO_4_-modified CAp HC scaffolds in saline (Otsuka Pharmaceutical, Tokyo, Japan) at 37 °C for 1, 4, 12, and 26 weeks, the remaining silver concentration in these scaffolds was quantified using ICP-AES.

The compressive strengths of the CAp HC scaffolds before and after Ag_3_PO_4_ modification and that of the clinically used 3D porous scaffolds (Osferion60-Marvelous) were measured using a universal testing machine (Autograph AGS-J, Shimadzu Corp., Kyoto, Japan). To measure the CAp HC scaffolds before and after Ag_3_PO_4_ modification, the scaffolds were compressed in a direction parallel to the channel direction. The crosshead speed was 1 mm/min, and eight samples were used for each scaffold.

### 2.4 In vitro cytotoxicity

The in vitro cytotoxicity of CAp HC scaffolds before and after Ag_3_PO_4_ modification were assayed via the 3-(4, 5-dimethylthiazol-2-yl)-2, 5-diphenyltetrazolium bromide (MTT) method using fibroblast-like cells (L929, JCRB9003, Japanese Collection of Research Bioresources Cell Bank, National Institutes of Biomedical Innovation, Health and Nutrition, Osaka, Japan) in accordance with the stipulations of ISO 10993–5 (Fig. 1B) [66]. Furthermore, considering that these scaffolds are used for bone regeneration, the in vitro cytotoxicity of the scaffolds was similarly evaluated using osteoblast-like cells (MC3T3-E1, RCB1126, Riken BioResource Center, Ibaraki, Japan), although they are not defined in ISO 10993–5. Positive (polyurethane film containing 0.1 % zinc diethyldithiocarbamate, RM-A, Lot No. A-212K) and negative (high-density polyethylene film, RM-C, Lot No. C-161) control materials were purchased from the Food and Drug Safety Center of the Hatano Research Institute (Kanagawa, Japan). The scaffolds were dry-heat sterilized at 170 °C for 3 h before use. The cells were cultured in Dulbecco's Modified Eagle's Medium (Fujifilm Wako Chemical) containing 10 % fetal bovine serum (Sigma-Aldrich, MO, USA) and 1 % penicillin-streptomycin (Fujifilm Wako Chemical) at 37 °C under 5 % CO_2_ for 24 h. The undiluted extract was designated as the 100 % extract. The 100 % extract was diluted with fresh medium to prepare the 50, 30, 10, and 3 % solutions. The MTT assay was performed using a thiazolyl blue tetrazolium bromide solution (Fujifilm Wako) in accordance with the instructions of the manufacturer. Six scaffolds from each group were used for the in vitro cytotoxicity assays.

To evaluate cell proliferation, attachment, and mineralization for Ag0-HC and Ag860-HC that achieved in vivo anti-infective bone formation without pigmentation, 3.5 × 10^4^ of MC3T3-E1 cells were seeded in each well in a 24-well plate (n = 9). Cells were fixed with 10 % formalin solution (Fujifilm Wako) after 24, 48, 72 h of incubation, and the cellular nuclei and actin were stained with Hoechst (Dojindo, Kumamoto, Japan) and red fluorescent dye-conjugated phalloidin (Acti-stain™ 555 phalloidin, Cytoskeleton, CO, USA), respectively. The cell numbers were counted using a fluorescence microscope with digital analysis software (BZ-X700, Keyence, Osaka, Japan). Calcified nodules with 10 % formalin solution after 14 days of cell incubation and stained with Alizarin Red S (ARS, Fujifilm Wako) to evaluated mineralization. ARS was extracted with 10 % (w/v) cetylpyridinium chloride (Tokyo Chemical Industry, Tokyo, Japan) buffer in 10 mM Na_2_HPO_4_ (Fujifilm Wako) overnight at 37 °C. Subsequently, 200 μL aliquots were transferred to a 96-well plate, and the absorbance at 560 nm was measured using a microplate reader (Multiskan FC).

### 2.5 In vitro genetic toxicity

When genetic toxicity is not observed at higher silver concentrations, it is considered to be not observed at lower silver concentrations. Therefore, the in vitro genetic toxicity of Ag2300-HC with the highest silver concentration that did not exhibit in vitro cytotoxicity was evaluated using the Ames test in accordance with the specifications of ISO 10993–3 [67]. Saline (Otsuka Pharmaceutical) was used as the negative control. 4-Nitroquinoline 1-oxide (4NQO), NaN_3_, and 2-aminoanthracene (2 A A) were purchased from Fujifilm Wako Chemicals. 9-Aminoacridine hydrochloride monohydrate (9 A A) was purchased from Sigma-Aldrich. 4NQO, 2 A A, 9 A A, and NaN_3_ were used as positive controls, and their concentrations are listed in Table S1. The saline extract liquid of Ag2300-HC (100 %) was diluted to prepare 1.56, 3.13, 6.25, 12.5, 25, and 50 % extract liquids, which were used as test solutions. The S9 mix (S-9/Cofactor A Set for Ames test, Oriental Yeast, Tokyo, Japan) was used as a metabolic activator. *Salmonella typhimurium* (TA100, TA1535, TA98, and TA1537) and *Escherichia coli* (WP2*uvrA*) were obtained from NITE.

Bacterial suspensions (1 × 10^9^ cells/mL, 0.1 mL) and 0.5 mL of 0.1 mol/L sodium phosphate buffer (pH 7.4, for test without the metabolic activation) or S9 mix (for test with the metabolic activation) were added to small test tubes (13 × 100 mm), which were subjected to high-pressure vapor sterilization (121 °C and 20 min). The test solutions (0.1 mL; 1.56, 3.13, 6.25, 12.5, 25, 50, and 100 % extract liquids) and positive control materials were added to the test tubes, which were then shaken at 37 °C for 20 min (amplitude: 2 cm; vibration: 120 cycles/min). Soft agar medium (2 mL) was added to the tubes, and the contents were poured onto a minimal glucose agar plate and spread uniformly by moving the plate. After fixing the agar medium, the plates were inverted and incubated at 37 °C for 48 h in an incubator.

Genetic toxicity was considered positive when the mean number of reversely mutated colonies per plate of any strain increased to more than twice that of the negative control and when the number of reversely mutated colonies increased with increasing doses of the test solution. Genetic toxicity was considered negative when the mean number of reversed mutant colonies in the test substance group was not more than twice that of the negative control group.

### 2.6 In vitro antibacterial activity

The in vitro antibacterial activities of the scaffolds were evaluated using the shaking and static culture methods established by the Society of Industrial Technology for Antimicrobial Articles and Grenho et al., respectively (Fig. 1B) [68,69]. The scaffolds were subjected to dry-heat sterilization at 170 °C for 3 h. The following bacteria were used: vancomycin-resistant enterococci (VRE; NCTC 12204, National Institute of Technology and Evaluation [NITE], Tokyo, Japan), *E. coli* (NBRC 3972, NITE), *Klebsiella pneumoniae* (NBRC 13277, NITE), *Pseudomonas aeruginosa* (NBRC 13275, NITE), *Staphylococcus aureus* subsp. *aureus* (NBRC 13276, NITE), methicillin-resistant *Staphylococcus aureus* (MRSA; IID 1677, International Research Center for Infectious Diseases, Institute of Medical Science, The University of Tokyo), *Staphylococcus epidermidis* (NBRC 12993, NITE), *Streptococcus mutans* (NBRC 13955, NITE). For the shaking method, each scaffold was immersed in a medium containing bacteria (1.0 × 10^5^ CFU/mL, 10 mL) using a sterilized lidded container and shaken horizontally at 150 rpm with an amplitude of 30 mm at 35 °C for 24 h after covering the container. For the static culture method, each scaffold was immersed in a medium containing bacteria (1.0 × 10^5^ CFU/mL, 10 mL) at 35 °C for 24 h. The number of viable bacteria in the test solution was evaluated using the plate culture method. The test solution (1 mL) was mixed with standard agar medium (9 mL), and the number of colonies was counted after incubation at 35 °C for 48 h. Antibacterial activity was calculated using the following equation:

R = Ut – At,

Where R is the antibacterial activity value and Ut and At are the means of the common logarithm of the number of viable bacteria for the control (Ag0-HC) and Ag_3_PO_4_-modified CAp scaffolds, respectively [70]. Thus, R values of 1 and 2 indicate 90 and 99 % reduction in bacteria, respectively.

### 2.7 In vivo acute systemic toxicity

The acute systemic toxicity was evaluated in accordance with the specifications of ISO 10993–11 to clarify the gap between in vitro cytotoxicity and in vivo toxicity and determine the Ag_3_PO_4_-modified CAp HC scaffold with the maximum silver content suitable for use in vivo bone formation evaluations (Fig. 1C) [71]. Extract liquids from Ag2300-HC, Ag5200-HC, and Ag21000-HC (0.2 g/mL) were prepared using saline and sesame oil at 121 °C for 1 h in accordance with the specifications of ISO 10993–12 [72]. Ag2300-HC, Ag5200-HC, and Ag21000-HC were subjected to dry-heat sterilization at 170 °C for 3 h. The test solutions (50 mL/kg) were intravenously and intraperitoneally injected into mice (3 weeks old, SLC:ICR, male, Japan SLC). Forty mice were used and divided into groups of five mice (Table S2). Their general conditions were observed at 10 min and 4, 24, 48, and 72 h after administration in accordance with the list shown in Table S3. Body weights were measured 24, 48, and 72 h after administration. After 72 h of observation, all mice were necropsied. The site of administration and major organs, including the heart, lungs, gastrointestinal tract, liver, spleen, kidneys, and genitals, were observed in the mice. The following criteria were used to determine the toxicity: (1) no acute systemic toxicity is judged to have occurred if all animals treated with the test solution do not show a stronger biological response than those in the control group throughout the observation period; (2) acute systemic toxicity is judged to be present when two or more animals in the test solution-treated group die or when two or more animals show significant toxic symptoms such as convulsions or weakness; (3) acute systemic toxicity is detected when three or more animals in the test solution group show a decrease in final body weight exceeding 10 % of the body weight at the time of administration; (4) if any of the following results are obtained, the test should be repeated (10 animals per group): (4–1) if any of the animals in the test solution group shows a minor biological response compared to that of the animals in the control group or (4–2) if a strong biological reaction or death is observed in only one animal.

### 2.8 In vivo anti-infective bone regeneration

Sixty-nine rabbits (male, Japanese white, 23 weeks old) were purchased from Japan SLC (Shizuoka, Japan) and randomly divided, as shown in Table 2. The experiments were initiated after a one-week acclimation period. The rabbits were individually reared with adequate amounts of a standard diet (LRC4, Oriental Yeast, Tokyo, Japan) and ad libitum water.

**Table 2:** **Analysis of bone regeneration in rabbits after implantation.** Percentages of rabbits with viable bacteria detected and findings of abscesses, osteonecrosis, and osteomyelitis at two and four weeks after implantation of clinically used 3D porous, Ag0-HC, Ag110-HC, Ag290-HC, and Ag860-HC scaffolds.

Scaffold	Time point (Weeks)	Number of animals	Viable bacteria detection (%)	Abscess incidence (%)	Osteonecrosis incidence (%)	Osteomyelitis incidence (%)
3D porous	2	6	50	66.7	16.7	0
	4	4	25	75	75	75
Ag0-HC	2	10	50	60	0	0
	4	10	30	30	30	0
Ag110-HC	2	5	20	40	0	0
	4	5	40	40	20	0
Ag290-HC	2	5	20	20	0	0
	4	5	20	40	0	0
Ag860-HC	2	9	0	0	0	0
	4	10	0	0	0	0

Animal experiments were conducted using a modified version of the method described by Yang et al. [73]. A mixed anesthetic drug (xylazine [5 mg/kg] and ketamine [30 mg/kg]) was intramuscularly administered for general anesthesia. Xylocaine (18 mg as lidocaine hydrochloride) was administered to the femoral muscle for local anesthesia. A cylindrical defect (diameter of 6 mm and depth of 4 mm) was created in the medial epicondyle of the femur using a trephine bar (outer diameter of 6 mm; Helmut Zepf, Land Baden-Württemberg, Germany). Subsequently, Ag0-HC, Ag110-HC, Ag290-HC, Ag860-HC, and clinically used 3D porous scaffolds (Osferion60-Marvelous) with a diameter of 6 mm and height of 4 mm were immersed in an MRSA-containing phosphate-buffered saline (PBS) solution (1 × 10^7^ CFU/mL) for 10 min and implanted into the femur bone defects. After rearing them for two and four weeks (Fig. 1D), the animals were euthanized using hyperanesthesia (intramuscular injection of 5 mg/kg xylazine and 30 mg/kg ketamine to sedate and subsequent intravenous injection of 10 mg/kg xylazine and 30 mg/kg ketamine) and the femoral condyles were collected for μ-CT imaging (ScanXmate-L090T) and histopathological analyses. The femoral condyles were fixed by immersing them in a 10 % neutral buffered formalin solution. After μ-CT scanning, the femoral condyle specimens were decalcified with a 0.5 mol/L ethylenediamine-N,N,N′,N′-tetraacetic acid tetrasodium salt tetrahydrate solution (Fujifilm Wako) at 30 °C for a month. Hematoxylin and eosin (HE) and Giemsa staining were performed after embedding the tissues in paraffin and sectioning them. Histopathological analysis was performed using a slide scanner and digital analysis software (Pannoramic DESKⅡ; 3DHISTECH, Budapest, Hungary).

To evaluate viable bacteria counts, the collected femoral condyles were weighed, rapidly frozen in liquid nitrogen, and pulverized into bone powder, according to previous reports [40,73]. The bone powder was sufficiently vortexed in PBS (10 mL) for 2 min, and the supernatant (50 μL) was collected and sequentially diluted 10°−10^9^-fold. Each diluted solution (0.1 mL) was smeared on X-MRSA agar medium (Nissui, Tokyo, Japan) and incubated at 35 °C for 24 h. After incubation, the number of colonies was counted to determine CFUs. The bacteria number in the bone powder was quantified, and values of fewer than 30 CFUs were rejected in accordance with AOAC international.

### 2.9 In vivo evaluation for bone formation, scaffold resorption, and pigmentation

Sixty rabbits (male, Japanese white, 18 weeks old, Japan SLC) were randomly divided into groups of five rabbits. The experiments were initiated after a one-week acclimation period. The rabbits were individually reared with adequate amounts of standard diet (LRC4) and water ad libitum.

A mixed anesthetic drug (xylazine [5 mg/kg] and ketamine [30 mg/kg]) was intramuscularly administered for general anesthesia. Xylocaine (18 mg as lidocaine hydrochloride) was administered to the femoral muscle for local anesthesia. A cylindrical defect (diameter of 6 mm and depth of 4 mm) was created in the medial epicondyle of the femur using a trephine bar (outer diameter of 6 mm; Helmut Zepf). Subsequently, Ag0-HC, Ag900-HC, Ag2300-HC, Ag5200-HC, Ag21000-HC, and clinically used 3D porous scaffolds (Osferion60 Marvelous) with a diameter of 6 mm and height of 4 mm were implanted into the femur bone defects (Fig. 1D). After rearing them for two and four weeks; the animals were euthanized using hyperanesthesia (intramuscular injection of 5 mg/kg xylazine and 30 mg/kg ketamine to sedate and subsequent intravenous injection of 10 mg/kg xylazine and 30 mg/kg ketamine). The femoral condyles were collected for μ-CT imaging (ScanXmate-L090T) and histological analysis. The femoral condyles were fixed by immersing them in a 10 % formalin neutral buffer solution. After μ-CT scanning, the femoral condyles underwent decalcification. After embedding them in paraffin and sectioning the femoral condyles, HE staining was performed. Histological analysis was performed using a slide scanner and digital analysis software (Pannoramic DESK II; 3DHISTECH).

### 2.10 Ethical statement

Animal experiments for in vivo anti-infective bone regeneration were approved by the Animal Care and Use Committee of the Japan SLC (approval nos. G03-0507 and G03-0560). Animal experiments for evaluating in vivo bone formation, scaffold resorption, and pigmentation were approved by the Animal Care and Use Committee of Kyushu University (approval no. A23-019-0). Acute systemic toxicity experiments were approved by the Animal Care and Use Committee of Japan SLC (approval no. G03-5197).

### 2.11 Statistical analysis

All statistical analyses were performed using EZR (Saitama Medical Center, Jichi Medical University, Saitama, Japan)—a graphical user interface for R (The R Foundation for Statistical Computing, Vienna, Austria). More precisely, it is a modified version of the R commander designed to add statistical functions frequently used in biostatistics [74]. All data are presented as the mean ± standard deviation, and *p*-values <0.05 are considered statistically significant. Statistical power was calculated for each analysis and confirmed (>80 %) in all the statistical tests. Normality was confirmed using the Kolmogorov–Smirnov test before all statistical tests. Comparisons between the two groups were performed using Student's t-test. Multiple comparisons were performed using the Tukey–Kramer test.

## 3 Results

### 3.1 Physicochemical, structural, and mechanical properties

CAp HC scaffolds were fabricated using the procedures described in our previous reports [65]. First, HC structures comprising a mixture of CaCO_3_ powder and an organic binder were prepared via extrusion molding. These HC structures were then heat-treated to remove the organic binder, resulting in CaCO_3_ HC structures. Finally, these structures were phosphatized by immersing them in an Na_2_HPO_4_ aqueous solution to convert the composition to CAp. Thus, CAp HC scaffolds were obtained.

To endow the CAp HC scaffolds with antibacterial activity, they were modified with Ag_3_PO_4_ via dissolution-precipitation reactions by immersing the scaffolds in various concentrations of AgNO_3_ aqueous solution at 20 °C for 1 h. The silver contents in the HC scaffolds after immersion were measured via inductively coupled plasma atomic emission spectrometry. The different concentrations of AgNO_3_, corresponding silver contents in the scaffolds, and their designated names are listed in Table 1.

**Fig. 2:**
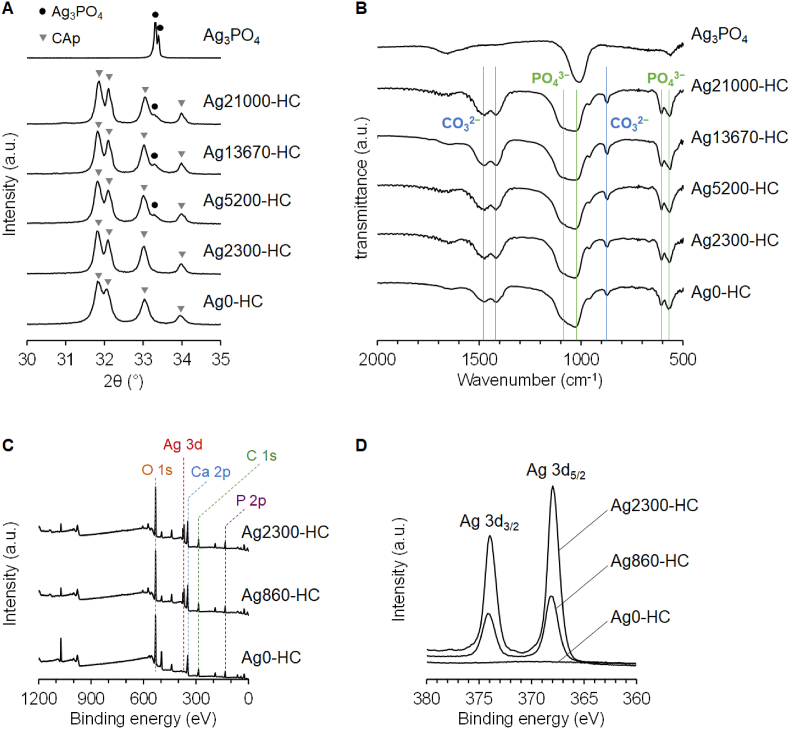
**Material characterization of CAp HC scaffolds**. (**A**) X-ray diffraction patterns and (**B**) Fourier transform infrared spectra of Ag0-HC, Ag2300-HC, Ag5200-HC, Ag13670-HC, and Ag21000-HC. X-ray photoelectron spectroscopy profiles of Ag0-HC, Ag 860-HC, and Ag2300-HC: (**C**) wide survey and (**D**) Ag 3d spectra.

XRD patterns indicate the presence of CAp in Ag0-HC (Fig. 2A). Although the diffraction peaks of CAp were detected in the XRD patterns of Ag110-HC, Ag480-HC, Ag860-HC, and Ag2300-HC, those of Ag_3_PO_4_ were not detected because of the detection limit of the XRD (Fig. 2A and S1A). The diffraction peaks of Ag_3_PO_4_ can be observed in the XRD patterns of Ag5200-HC, Ag13670-HC, and Ag21000-HC. In the FTIR spectra of the various HC scaffolds, absorption bands of apatitic phosphate were detected at 1098, 1029, 604, and 563 cm^−1^, and those of carbonate were detected at 1473, 1414, and 869 cm^−1^ (Fig. 2B and S1B) [75]. The bands of Ag_3_PO_4_ were not clearly observed because they overlapped with those of apatitic phosphate (Fig. 2B and S1B). In the XPS profiles of Ag860-HC and Ag2300-HC, the Ag 3d_3/2_ and 3d_5/2_ peaks were detected at 373.8 and 367.8 eV, respectively (Fig. 2C and D), which coincided with the value of Ag^+^ in Ag_3_PO_4_ [76]. Thus, the XPS profiles indicated Ag_3_PO_4_ formation on the scaffold surfaces, although the diffraction peaks of Ag_3_PO_4_ were not detected in the XRD patterns. Furthermore, no N 1S peak was detected in the XPS profiles of Ag860-HC and Ag2300-HC (Fig. 2C). CHN elemental analysis demonstrated that none of the HC scaffolds contained nitrogen. Thus, no AgNO_3_ remained in HC scaffolds after Ag_3_PO_4_ modification. Furthermore, all HC scaffolds contained 10–11 % carbonate derived from CAp.

**Fig. 3:**
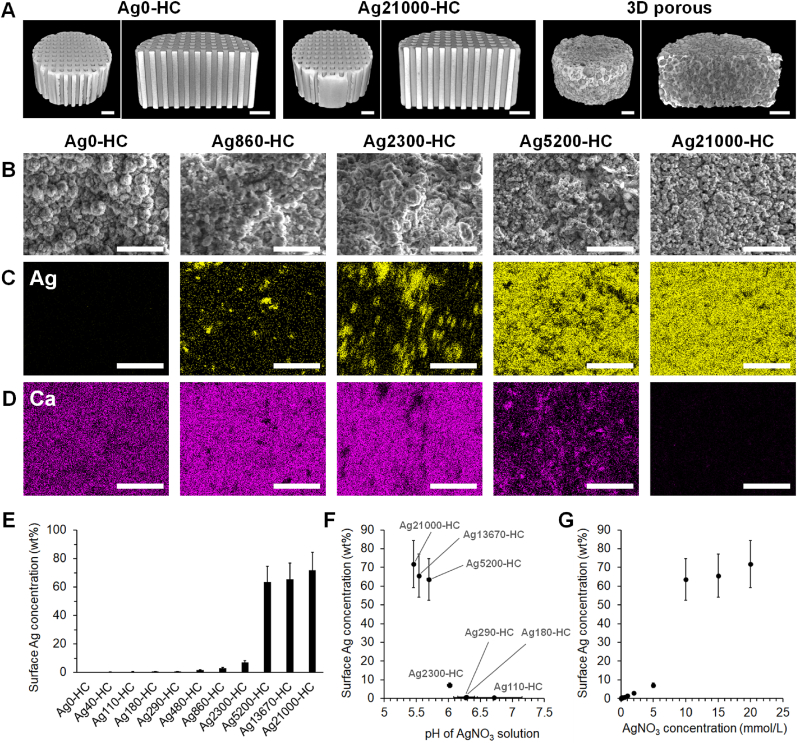
**Structural analysis and morphological characterization of CAp HC scaffolds and effect of pH and concentration of the AgNO**_**3**_**solution on surface silver concentration**. (**A**) Micro-computed tomography (μ-CT) images of Ag0-HC, Ag21000-HC, and the clinically used 3D porous scaffolds. Scale bar: 1 mm. (**B**) Scanning electron microscopy images and energy dispersive X-ray maps of (**C**) Ag and (**D**) Ca of Ag0-HC, Ag860-HC, Ag2300-HC, Ag5200HC, and Ag21000-HC. Scale bar: 20 μm. (**E**) Surface silver concentrations of Ag0-HC, Ag40-HC, Ag110-HC, Ag180-HC, Ag290-HC, Ag480-HC, Ag860-HC, Ag2300-HC, Ag5200HC, Ag13670HC, and Ag21000-HC. (**F**) Correlation between the surface silver concentration and pH of the AgNO_3_ solution. (**G**) Correlation between the surface silver concentration and concentration of the AgNO_3_ solution.

μ-CT images showed that Ag0-HC and Ag21000-HC possessed HC structures with many channels that uniaxially penetrated the scaffold (Fig. 3A). Thus, the HC structure was maintained even after Ag_3_PO_4_ modification by immersion in the AgNO_3_ solution with the highest concentration. The clinically used 3D porous scaffolds (Osferion60-Marvelous, Olympus Terumo Biomaterials), which were used as controls, contained numerous spherical pores (Fig. 3A). However, most of the pores were not completely interconnected and were present as closed pores. SEM images (Fig. 3B and S2A) and elemental maps of silver (Fig. 3C and S2B) and calcium (Fig. 3D and S2C) on the HC scaffold surfaces demonstrated that the surface silver concentration increased while the Ca concentration decreased with increasing AgNO_3_ concentration, indicating that Ag_3_PO_4_ replaced CAp on the surface regions of the HC scaffolds. Surface silver concentrations in Ag0-HC, Ag40-HC, Ag110-HC, Ag180-HC, Ag290-HC, Ag480-HC, Ag860-HC, Ag2300-HC, Ag5200-HC, Ag13670-HC, and Ag21000-HC—which were measured via EDX analysis—were 0.00 ± 0.00, 0.25 ± 0.04, 0.43 ± 0.08, 0.52 ± 0.09, 0.67 ± 0.12, 1.52 ± 0.27, 2.87 ± 0.50, 7.07 ± 1.23, 63.5 ± 11.1, 65.5 ± 11.4, and 71.9 ± 12.6 wt%, respectively (Fig. 3E). Thus, the surface silver concentrations in the HC scaffolds were significantly higher than the silver content in the entire HC scaffold (Table 1), demonstrating the localization of Ag_3_PO_4_ on the surfaces of the HC scaffolds. Furthermore, the surface silver concentration drastically changed between Ag2300-HC and Ag5200-HC (Fig. 3E), which correlated with the pH of the AgNO_3_ solution (Fig. 3F). Thus, the surface silver concentration differed when the pH values were greater and less than 6 (Fig. 3F). A large change in the surface silver concentration occurred between Ag2300-HC (pH = 6.0) and Ag5200-HC (pH = 5.7). Moreover, in AgNO_3_ solution concentrations of ≤5 mmol/L (pH ≥ 6), the surface silver concentration increased linearly with increasing AgNO_3_ concentration (Fig. 3G).

**Fig. 4:**
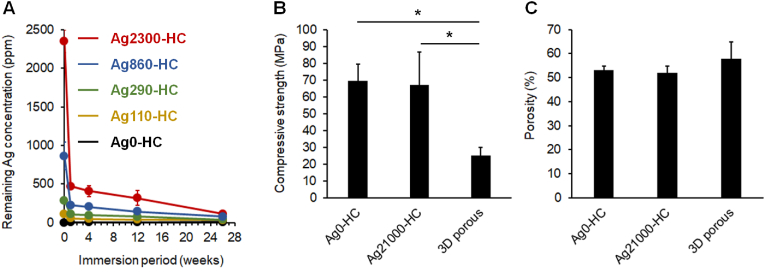
**Analysis of remaining silver concentration, compressive strength, and porosity**. (**A**) Remaining silver concentration after immersion in physiological saline at 37 °C. (**B**) Compressive strengths and (**C**) porosities of Ag0-HC, Ag21000-HC, and 3D porous scaffolds. *p < 0.05.

The remaining silver concentrations in Ag0-HC, Ag110-HC, Ag290-HC, Ag860-HC, and Ag2300-HC after immersion in physiological saline at 37 °C were evaluated for up to 26 weeks (Fig. 4A). The silver content decreased drastically during the first week and then gradually decreased until week 26. The compressive strength of Ag0-HC (69.5 ± 10.0 MPa) was almost equal to that of Ag21000-HC (67.2 ± 19.7 MPa), indicating that the mechanical strength of CAp HC scaffolds was maintained after Ag_3_PO_4_ modification (Fig. 4B). The compressive strengths of Ag0-HC and Ag21000-HC were significantly higher than that of the clinically used 3D porous scaffold (25.1 ± 5.1 MPa). Furthermore, the porosities of the Ag0-HC, Ag21000-HC, and 3D porous scaffolds were 53.2 ± 1.5, 51.9 ± 2.9, and 57.9 ± 7.1 %, respectively (Fig. 4C). Thus, no significant differences in porosity were observed between these scaffolds. Therefore, the HC scaffolds exhibited superior mechanical properties to those of clinically used 3D porous scaffolds, although their porosities were similar.

### 3.2 In vitro cytotoxicity

**Fig. 5:**
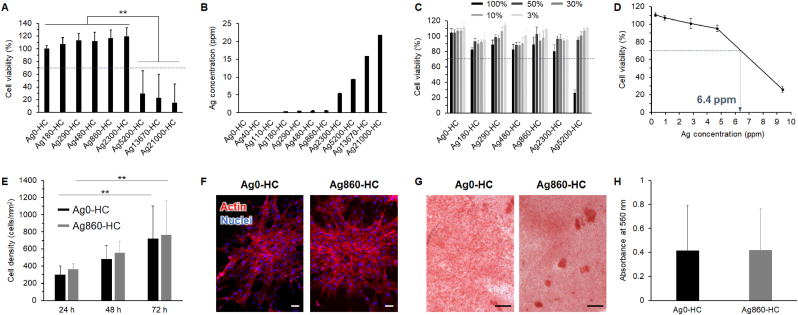
**Analysis of in vitro cytotoxicity of various CAp HC scaffolds**. (**A**) Cell viability of Ag0-HC, Ag180-HC, Ag290-HC, Ag480-HC, Ag860-HC, Ag2300-HC, Ag5200-HC, Ag13670-HC, and Ag21000-HC for MC3T3-E1 cells. The dotted line indicates 70 % cell viability. Multiple comparisons were performed using the Tukey–Kramer test (**p < 0.01). (**B**) Silver concentration in culture media of Ag0-HC, Ag40-HC, Ag110-HC, Ag180-HC, Ag290-HC, Ag480-HC, Ag860-HC, Ag2300-HC, Ag5200-HC, Ag13670-HC, and Ag21000-HC. (**C**) Cell viability of the extracts from Ag0-HC, Ag180-HC, Ag290-HC, Ag480-HC, Ag860-HC, Ag2300-HC, and Ag5200-HC for L929 cells. Undiluted extract (100 % extract) and those diluted with culture medium to concentrations of 50, 30, 10, and 3 % of the extract concentration were used. The dotted line indicates 70 % cell viability. (**D**) Correlation between cytotoxicity and silver concentration in the culture medium. The dotted line indicates the Ag concentration when cell viability is 70 %. Sample numbers per group are six for all in vitro cytotoxicity tests. (**E**) Proliferation of MC3T3-E1 cells in the Ag0-HC and Ag860-HC groups. Cell densities at 24, 48, and 72 h of incubation (**p < 0.01). (**F**) Fluorescent images of MC3T3-E1 cells with actin filaments and cellular nuclei stained with red fluorescent dye-conjugated phalloidin and Hoechst, respectively, after incubation of 72 h. Scale bar: 50 μm. (**G**) Calcified nodules stained with Alizarin Red S after 14 days of MC3T3-E1 cell incubation. Scale bar: 100 μm. (**H**) The absorbance of Alizarin Red S extraction liquids at 560 nm. (For interpretation of the references to colour in this figure legend, the reader is referred to the Web version of this article.)

The in vitro cytotoxicity of the various CAp HC scaffolds was evaluated in osteoblast- (MC3T3-E1 cells) and fibroblast-like cells (L929 cells) using MTT assay. When the cell viability is less than 70 %, the scaffold is considered cytotoxic in accordance with the stipulations of the International Organization for Standardization (ISO) standard 10,993–5 [66]. The viability of MC3T3-E1 cells in Ag0-HC, Ag180-HC, Ag290-HC, Ag480-HC, Ag860-HC, and Ag2300-HC was >70 % and that in Ag5200-HC, Ag13670-HC, and Ag21000-HC was <70 % (Fig. 5A). No cytotoxicity was observed in Ag0-HC, Ag180-HC, Ag290-HC, Ag480-HC, Ag860-HC, and Ag2300-HC. Furthermore, silver concentrations in the culture media of Ag2300-HC and Ag5200-HC were 5.5 ± 0.1 and 9.4 ± 0.1 mg/L, respectively, demonstrating the lack of cytotoxicity at silver concentrations below approximately 5.5 mg/L (Fig. 5B). Cytotoxicity was evaluated using L929 cells—which are more sensitive than MC3T3-E1 cells—at different dilution rates of Ag0-HC, Ag180-HC, Ag290-HC, Ag480-HC, Ag860-HC, Ag2300-HC, and Ag5200-HC extracts (Fig. 5C and Table S4). When undiluted extract (100 % extract) was used, no toxicity was observed for L929 cells in Ag0-HC, Ag180-HC, Ag290-HC, Ag480-HC, Ag860-HC, and Ag2300-HC, whereas toxicity was observed in Ag5200-HC (Fig. 5C), in addition to the cytotoxicity results for MC3T3-E1 (Fig. 5A). No cytotoxicity was detected for L929 cells in the extracts diluted with the culture medium to concentrations of 50, 30, 10, and 3 % of the extract concentration (Fig. 5C). From the plot of cell viability versus silver concentration in the culture medium, cytotoxicity to L929 cells was observed at 6.4 ppm of silver concentration (Fig. 5D).

MC3T3-E1 cells proliferated during the incubation period of 24–72 h in the Ag0-HC and Ag860-HC groups (Fig. 5E). Fluorescent images showed that the actin filaments developed after 72 h of incubation in the Ag0-HC and Ag860-HC groups (Fig. 5F). After 14 days of MC3T3-E1 cell incubation, calcified nodules were stained with ARS in the Ag0-HC and Ag860-HC groups (Fig. 5G). No significant difference was detected in the absorbance of ARS extraction liquids at 560 nm between the Ag0-HC and Ag860-HC groups (Fig. 5H). Thus, Ag_3_PO_4_ modification of CAp HC scaffolds did not prevent cell proliferation and mineralization.

### 3.3 In vitro genetic toxicities

The genetic toxicity of Ag2300-HC with the maximum silver content that did not exhibit cytotoxicity was evaluated with a reverse mutation assay using *Salmonella typhimurium* (TA100, TA1535, TA98, and TA1537) and *E. coli* (strain: WP2 *uvrA*) in accordance with the specifications of ISO10993-3 [66]. For all the strains, the colony count in the Ag2300-HC group did not increase to more than twice that in the negative control group (Table S5 and Fig. S3). Therefore, Ag2300-HC exhibited no genetic toxicity.

### 3.4 In vitro antibacterial activity

**Fig. 6:**
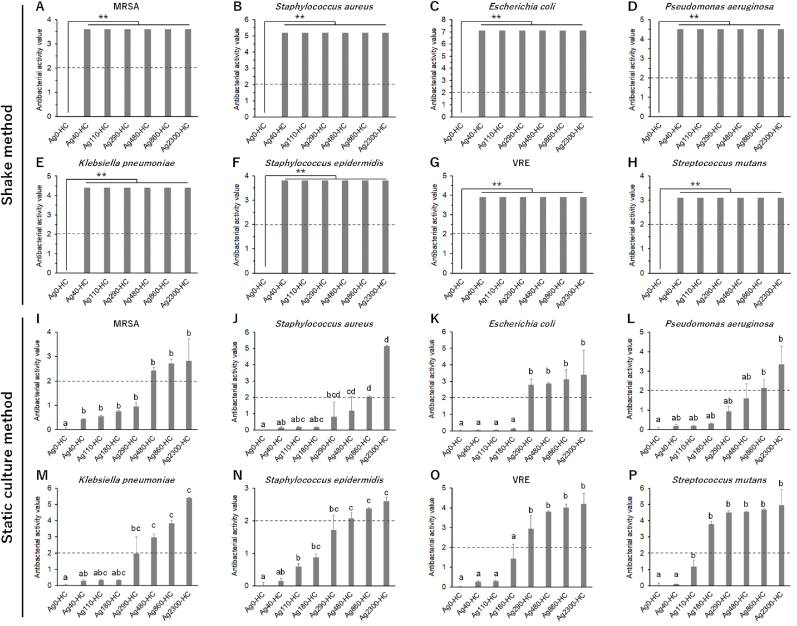
**Antibacterial activity values Ag0-HC, Ag40-HC, Ag110-HC, Ag290-HC, Ag480-HC, Ag860-HC, and Ag2300-HC for various types of bacteria under (A–H) shaking and (I–P) static conditions**. Bacteria type: (**A**, **I**) Methicillin-resistant *Staphylococcus aureus*, (**B**, **J**) *Staphylococcus aureus*, (**C**, **K**) *Escherichia coli*, (**D**, **L**) *Pseudomonas aeruginosa*, (**E**, **M**) *Klebsiella pneumoniae*, (**F**, **N**) *Staphylococcus epidermidis*, (**G**, **O**) vancomycin-resistant *enterococci*, and (**H**, **P**) *Streptococcus mutans*. Sample numbers per group are six for all in vitro antibacterial tests. The dotted lines in all figures indicate an antibacterial activity value of 2. Multiple comparisons were performed using the Tukey–Kramer test. **(A**–H) **p < 0.01. **(I–P)** Different letters (a, b, c, and d) indicate statistically significant differences among groups (*p* < 0.05).

The in vitro antibacterial activities of the HC scaffolds that did not exhibit cytotoxicity (Ag0-HC, Ag40-HC, Ag110-HC, Ag290-HC, Ag480-HC, Ag860-HC, and Ag2300-HC) for various types of bacteria were evaluated under shaking and static conditions. Ag40-HC, Ag110-HC, Ag290-HC, Ag480-HC, Ag860-HC, and Ag2300-HC exhibited antibacterial activity values higher than 2, i.e., 99 % bacterial lethality, for MRSA, *Staphylococcus aureus, Escherichia coli* (*E. coli*), *Pseudomonas aeruginosa*, *Klebsiella pneumoniae*, *Staphylococcus epidermidis,* VRE, and *Streptococcus mutans* under shaking conditions (Fig. 6A–H). Ag0-HC did not exhibit antibacterial activity against any bacteria (Fig. 6A–H). Under static conditions, Ag860-HC and Ag2300-HC exhibited antibacterial activity values greater than 2 for all bacteria (Fig. 6I–P). Ag290-HC and Ag480-HC exhibited antibacterial activity values higher than 1, that is, 90 % bacterial lethality, for all bacteria (Fig. 6I–P). The antibacterial activity values of Ag40-HC and Ag110-HC were higher than 0 and lower than 1, whereas that of Ag0-HC was 0 (Fig. 6I–P). Thus, Ag860-HC and Ag2300-HC exhibited both non-cytotoxicity and antibacterial activity against various types of bacteria (broad antibacterial spectra) under all conditions.

### 3.5 In vivo acute systemic toxicity

In vivo acute systemic toxicities of not only with Ag2300-HC that had the highest silver content in the range of no in vitro cytotoxicity but also with Ag5200-HC and Ag21000-HC that exhibit in vitro cytotoxicity were evaluated to clarify the gap between in vitro cytotoxicity and in vivo toxicity and determine the upper limit of acceptable silver concentrations in vivo. Ag13670-HC was not evaluated because when Ag21000-HC with the maximum silver content is not toxic, scaffolds with lower silver content are not considered toxic. In vivo acute systemic toxicities of Ag2300-HC, Ag5200-HC, and Ag21000-HC were evaluated in accordance with the stipulations of ISO10993-11 [71]. Extract liquids were prepared using saline and sesame oil at 121 °C for 1 h, in accordance with the specifications of ISO10993-12 [72].

None of the animals showed clinical or anatomic abnormalities, gross lesions, reduction in body weight, or death at 10 min and 4, 24, 48, and 72 h after intraperitoneal and intravenous injections of the extract liquids in all groups (Tables S5–S8 and Fig. S4). Thus, although Ag5200-HC and Ag21000-HC exhibited in vitro cytotoxicity, they did not exhibit in vivo acute systemic toxicity, demonstrating that all Ag_3_PO_4_-modified CAp HC scaffolds with silver content lower than Ag21000-HC can be used for in vivo bone formation evaluations.

### 3.6 In vivo anti-infective bone regeneration in the presence of bacteria

**Fig. 7:**
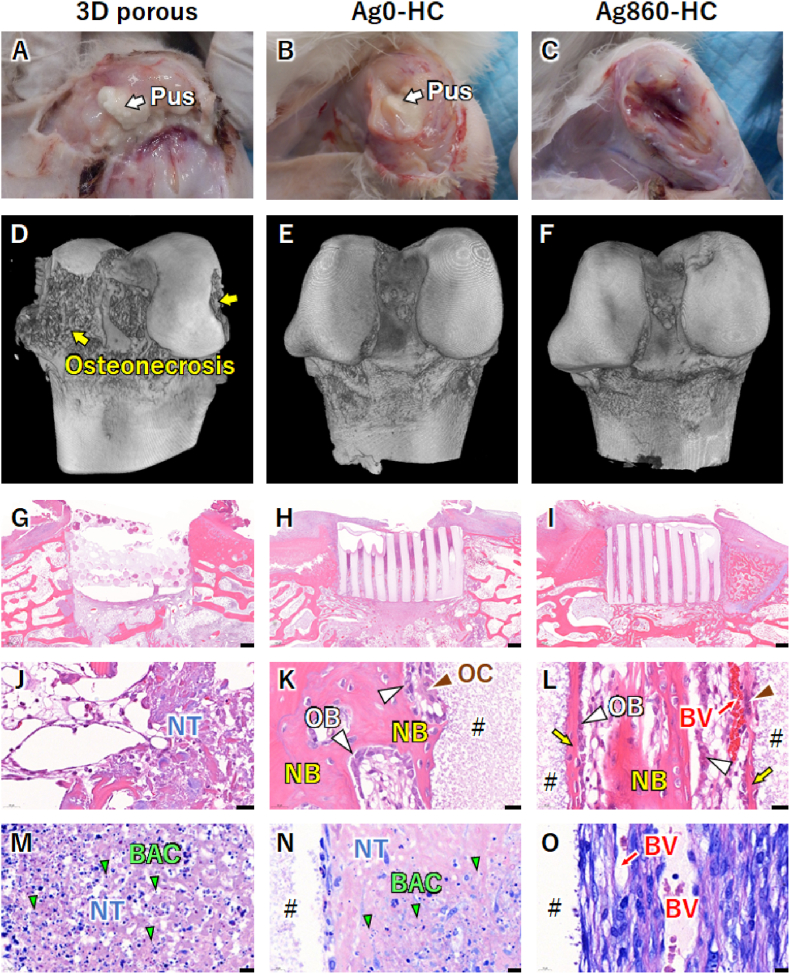
**In vivo results on anti-infective bone regeneration at two weeks after implantation**. Images of femoral regions with skin incision in (**A**) clinically used 3D-porous-scaffold-, (**B**) Ag0-HC-, and (**C**) Ag860-HC-implanted groups. μ-CT images of rabbit femurs in (**D**) 3D-porous-scaffold-, (**E**) Ag0-HC-, and (**F**) Ag860-HC-implanted groups. The yellow arrow indicates osteonecrosis. Hematoxylin and eosin (HE)-stained histological sections of (**G**) 3D-porous-scaffold-, (**H**) Ag0-HC-, and (**I**) Ag860-HC-implanted groups. (**J–L**) High-magnification HE-stained images. Giemsa-stained histological sections of (**M**) 3D-porous-scaffold-, (**N**) Ag0-HC-, and (**O**) Ag860-HC-implanted groups. Scale bars: (**G–I**) 500 μm, (**J–L**) 20 μm, and (**M–O**) 10 μm. NB and yellow arrows indicate new bones. BV and red arrows indicate blood vessels. OB and white arrow heads indicate osteoblasts. OC and blown arrowheads indicate osteoclasts. #, NT, and BAC indicate the remaining material, necrotic tissue, and bacteria, respectively. (For interpretation of the references to colour in this figure legend, the reader is referred to the Web version of this article.)

Clinically used 3D porous scaffolds, Ag0-HC, Ag110-HC, Ag290-HC, and Ag860-HC were individually immersed in MRSA solutions (1 × 10^7^ CFU/mL, 2 mL) for 10 min and subsequently implanted into bone defects (6 mm in diameter and 5 mm in depth) in rabbit femur condyles to clarify the minimum effective silver concentration and structural effects for in vivo anti-infective bone regeneration. Two weeks after implantation, viable bacteria were detected and abscesses were formed in the 3D-porous-scaffold-, Ag0-HC-, Ag110-HC-, Ag290-implanted groups (Table 2 and Fig. 7 and S5). Notably, the collapse of femur condyles, that is, osteonecrosis, was observed in only the 3D-porous-scaffold-implanted group (Fig. 7D), indicating that the infection symptoms in the 3D-porous-scaffold-implanted group were more severe than those in other groups (Fig. 7E and F, S5C, and S5D). The percentages of viable bacteria detection and abscess incidence decreased with increasing silver concentration (Table 2). In the Ag860-implanted group, no viable bacteria were detected and no abscess, osteonecrosis, and osteomyelitis were observed (Table 2 and Fig. 7C–F). HE staining showed that minimal tissues were formed in the 3D porous scaffold (Fig. 7G), whereas abundant tissues were formed in the channels of Ag0-HC-, Ag110-HC-, Ag290-HC-, and Ag860-HC scaffolds (Fig. 7H and I, S5E, and S5F). High-magnification HE images showed that the tissues surrounding the 3D porous scaffold were necrotic (Fig. 7J). In contrast, new bones were formed on the surfaces and in the interiors of Ag0-HC, Ag110-HC, Ag290-HC, and Ag860-HC (Fig. 7K and L, S5G, and S5H). Furthermore, blood vessels were observed near the new bones, and osteoblasts and osteoclasts resided in the new bones and scaffold, respectively (Fig. 7K and L, S5G, and S5H). Giemsa staining revealed that numerous bacteria were present in the necrotic tissues around the 3D porous scaffolds (Fig. 7M). In the Ag0-HC-implanted group, bacteria and necrotic tissue were observed at the periosteal ends of the scaffold channels (Fig. 7N). A small number of bacteria was observed in the Ag110-HC- and Ag290-HC-implanted groups (Figs. S5I and S5J), while no bacteria were detected in the Ag860-HC-implanted group (Fig. 7O).

**Fig. 8:**
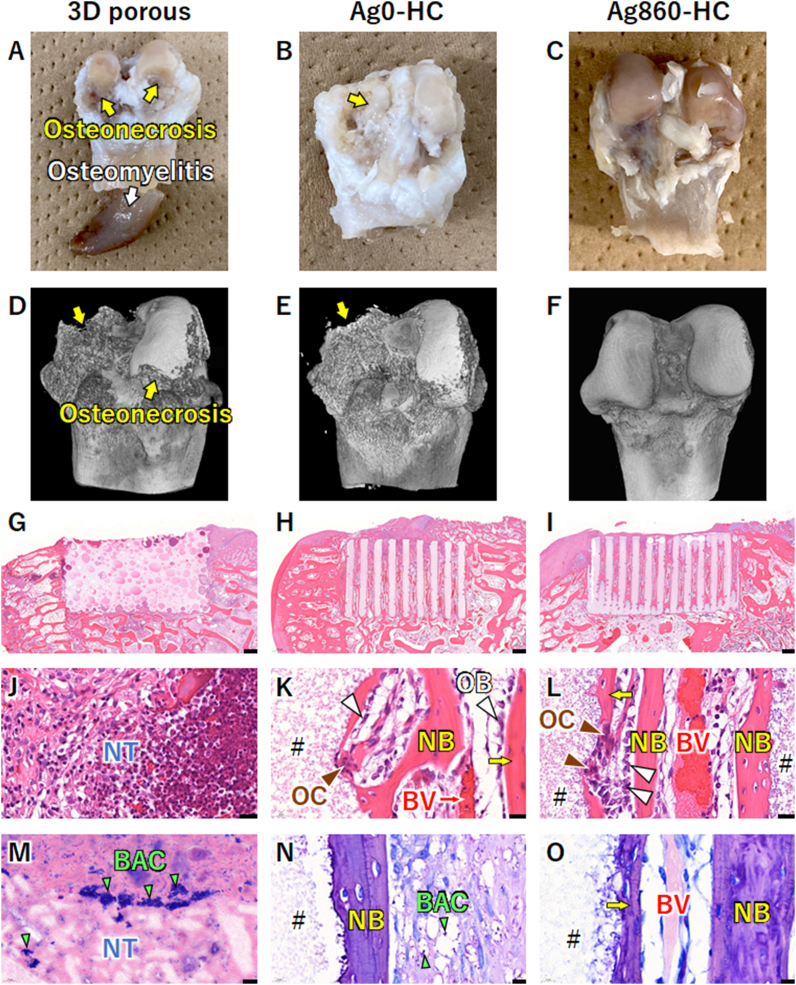
**In vivo results on anti-infective bone regeneration at two weeks after implantation**. Images of femoral regions with skin incision in (**A**) clinically used 3D-porous-scaffold-, (**B**) Ag0-HC-, and (**C**) Ag860-HC-implanted groups. μ-CT images of rabbit femurs in (**D**) 3D-porous-scaffold-, (**E**) Ag0-HC-, and (**F**) Ag860-HC-implanted groups. The yellow and white arrows indicate osteonecrosis and osteomyelitis, respectively. HE-stained histological sections of (**G**) 3D-porous-scaffold-, (**H**) Ag0-HC-, and (**I**) Ag860-HC-implanted groups. (**J–L**) High-magnification HE-stained images. Giemsa-stained histological sections of (**M**) 3D-porous-scaffold-, (**N**) Ag0-HC-, and (**O**) Ag860-HC-implanted groups. Scale bars: (**G–I**) 500 μm, (**J–L**) 20 μm, and (**M–O**) 10 μm. NB and yellow arrows indicate new bones. BV and red arrows indicate blood vessels. OB and white arrow heads indicate osteoblasts. OC and blown arrowheads indicate osteoclasts. #, NT, and BAC indicate the remaining material, necrotic tissue, and bacteria, respectively. (For interpretation of the references to colour in this figure legend, the reader is referred to the Web version of this article.)

Four weeks after the implantation of clinically used 3D porous scaffolds, abscesses, osteomyelitis, and osteonecrosis occurred in 75 % of rabbits, which were higher than the percentages of these symptom incidences in the Ag0-HC-implanted group (Table 2 and Fig. 8 and S6). Abscess and osteonecrosis incidence decreased with increasing silver concentration (Table 2). In the Ag860-HC-implanted groups, no abscess and osteonecrosis occurred (Table 2 and Fig. 8 and S6). No osteomyelitis findings were reported in the HC scaffold groups with and without silver phosphate (Table 2 and Fig. 8 and S6). μ-CT images clearly showed osteonecrosis in the 3D-porous-scaffold-, Ag0-HC-, and Ag110-HC-implanted groups, whereas no osteonecrosis was observed in the Ag290-HC- and Ag860-HC-implanted groups (Table 2 and Fig. 8F and S6D). HE staining showed that only minimal bone formation occurred in the interior of the 3D porous scaffolds, even after four weeks of implantation (Fig. 8G). In contrast, new bones were abundantly formed throughout the scaffold channels in the Ag0-HC-, Ag110-HC-, Ag290-HC-, and Ag860-HC-implanted groups (Fig. 8H and I, S6E, and 86F). High-magnification images demonstrated that necrotic tissues remained around the 3D porous scaffolds (Fig. 8J). In contrast, new bone and blood vessels were formed in the scaffold channels in the Ag0-HC-, Ag110-HC-, Ag290-HC-, and Ag860-HC-implanted groups (Fig. 8K and L, S6G, and S6H). Furthermore, osteoblasts were arranged on the new bone, osteoclasts resided on the HC scaffolds, and resorption lacunae were formed (Fig. 8K and L, S6G, and S6H). Giemsa staining revealed bacterial aggregates in the necrotic tissues around the 3D porous scaffolds (Fig. 8M). In the Ag0-HC-, Ag110-HC-, and Ag290-HC-implanted groups, a small number of bacteria were observed in some specimens (Fig. 8N–S6I, and S6J). No bacteria were observed in the Ag860-HC-implanted groups (Fig. 8O).

### 3.7 In vivo bone regeneration without pigmentation

**Fig. 9:**
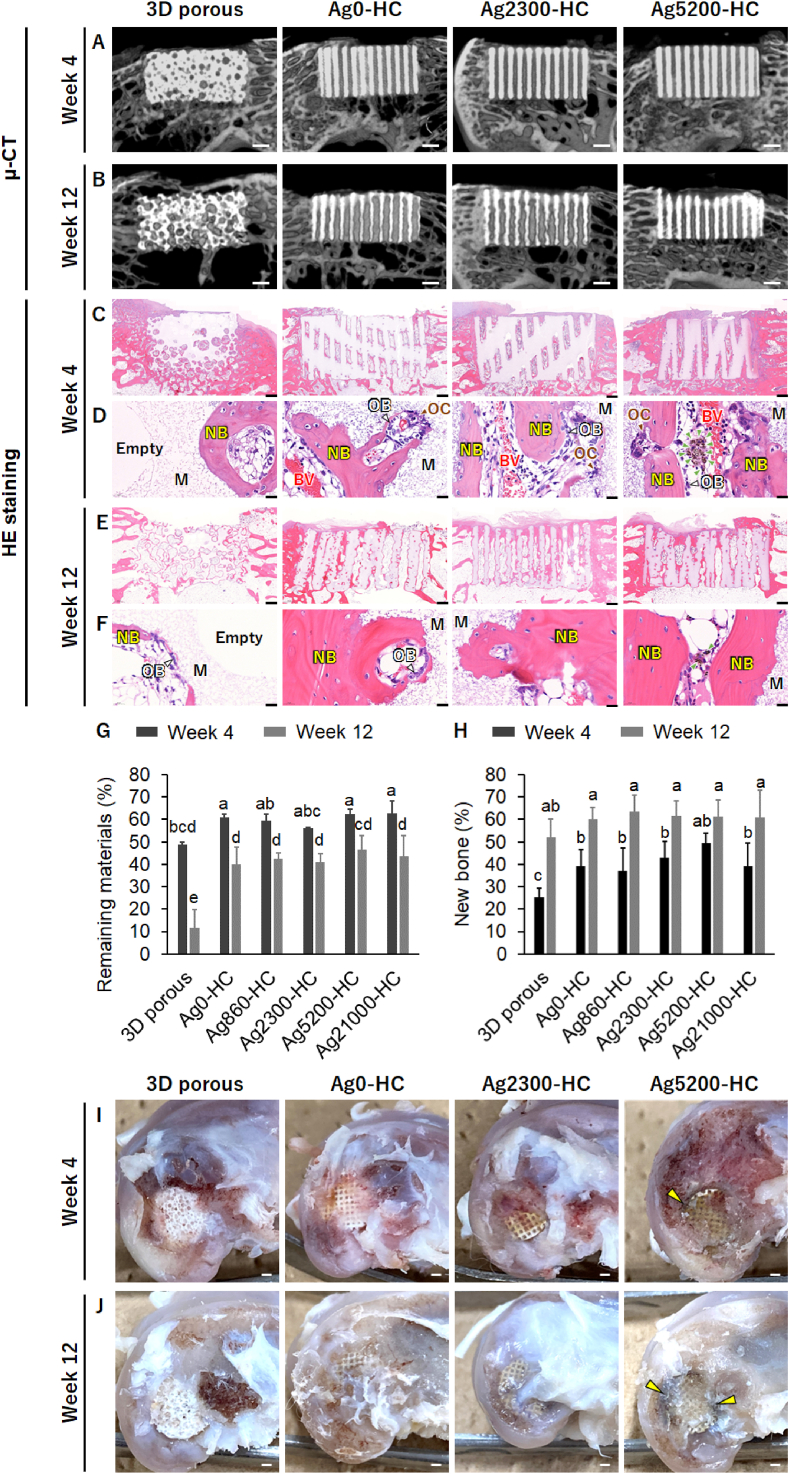
**Analysis of in vivo bone regeneration and pigmentation for different scaffolds at weeks 4 and 12**. μ-CT images of 3D porous, Ag0-HC, Ag2300-HC, and Ag5200-HC scaffolds at weeks (**A**) 4 and (**B**) week 12 after implantation. HE staining of 3D porous, Ag0-HC, Ag2300-HC, and Ag5200-HC scaffolds at weeks (**C**) 4 and (**D**) 12 after implantation. #, Empty, NB, BV, OB, and OC indicate the remaining material, empty pores, new bone, blood vessels, osteoblasts, and osteoclasts, respectively. Green arrowheads indicate brown-stained giant cells, which are considered macrophages that phagocytize silver. (**G**) Percentages of remaining materials at 4 and 12 weeks after implantation. Different letters (a, b, c, d, and e) indicate statistically significant differences among groups (*p* < 0.05). (**H**) Percentages of newly formed bone in the scaffolds at 4 and 12 weeks after implantation. Different letters (a and b) indicate statistically significant differences among groups (*p* < 0.05). Gross anatomical findings at weeks (**I**) 4 and (J) 12. Yellow arrowheads indicate pigmented regions. Scale bars: (**A, B**) 1 mm, (**C**, **E**) 500 μm, (**D**, **F**) 20 μm, and (**I, J**) 1 mm. (For interpretation of the references to colour in this figure legend, the reader is referred to the Web version of this article.)

Ag0-HC, Ag860-HC, Ag2300-HC, Ag5200-HC, and Ag21000-HC were implanted into bone defects in the rabbit femur condyles under aseptic conditions to clarify the effects of silver concentration on bone formation and material resorption and the maximum silver concentration that causes no pigmentation. Furthermore, the adequacy of these HC scaffolds for clinical use was verified by comparing the bone formation on clinically used 3D porous scaffolds. μ-CT images showed that all grafts gradually resorbed from week 4 (Fig. 9A and S7A) to week 12 (Fig. 9B and S7B). HE staining demonstrated that no tissues were formed within several pores, and empty pores were present in the 3D porous scaffolds at week 4, although new bones were formed in some pores (Fig. 9C–D). In contrast, new bones and blood vessels were formed within all channels in the Ag0-HC, Ag860-HC, Ag2300-HC, Ag5200-HC, and Ag21000-HC groups (Fig. 9C and D, S7C, and S7D). Furthermore, osteoblasts and osteoclasts resided on the new bones and in the resorption lacunae formed in the struts of these HC scaffolds (Fig. 9C and D, S7C, and S7D). In Ag5200-HC and Ag21000-HC, brown-stained giant cells, considered macrophages that phagocytized silver, were observed in the channels (Fig. 9C and D, S7C, and S7D). At week 12, new bones did not form within all pores in the 3D porous scaffolds, and empty pores remained (Fig. 9E–F). Conversely, abundant bones were observed in all the channels of Ag0-HC, Ag860-HC, Ag2300-HC, Ag5200-HC, and Ag21000-HC (Fig. 9E and F, S7E, and S7F). Furthermore, the struts of these HC scaffolds were partially resorbed, and the resorbed portions were filled with new bones (Fig. 9E and F, S7E, and S7F). In Ag5200-HC and Ag21000-HC, giant cells were considered as macrophages that phagocytized the silver maintained in the channels (Fig. 9E and F, S7E, and S7F). The percentage of the remaining material of the 3D porous scaffolds was lower than those of the HC scaffolds at weeks 4 and 12 (Fig. 9G). No statistically significant difference was detected in the percentages of the remaining materials between the HC scaffolds. The percentages of the remaining materials significantly decreased between weeks 4 and 12 in all scaffolds (Fig. 9G). The 3D porous and HC scaffolds exhibited approximately 37 % and 15–20 % decrease in the percentages of the remaining materials between weeks 4 and 12, respectively (Fig. 9G). Thus, the 3D porous scaffolds were resorbed approximately twice as quickly as the HC scaffolds. However, the percentage of new bones at week 4 in the 3D porous scaffolds (25.1 ± 4.4 %) was significantly lower than those in all the HC scaffolds (37–50 %, Fig. 9H). Notably, Ag5200-HC (49.9 ± 4.2 %) exhibited a higher percentage of new bones than the other HC scaffolds (37–43 %, Fig. 9H). At week 12, no statistically significant difference in the new bone percentage was observed between all the scaffolds (Fig. 9H). The images showed that no pigmentation occurred in 3D-porous-, Ag0-HC-, Ag860-HC-, and Ag2300-HC-implanted groups at weeks 4 and 12 (Fig. 9I and J, S7G, and S7H). However, black pigmentation on and around the scaffolds in the Ag5200-HC- and Ag21000-HC-implanted groups was observed at week 4 (Fig. 9I and S7G), which persisted till week 12 (Fig. 9J and S7H).

## 4 Discussion

In this study, we established a method to modify CAp scaffolds with 11 different concentrations of Ag_3_PO_4_ and determined the optimum silver concentration in terms of anti-infection, bone regeneration, toxicity, and pigmentation, as well as the differences in antibacterial activity and toxicity between in vitro and in vivo evaluations and between evaluation methods. The findings of our study suggest that the scaffold structure can achieve anti-infective effects.

Regarding Ag_3_PO_4_ modification, our modification method could localize Ag_3_PO_4_ on the surface of CAp HC scaffolds, which allowed the scaffold to release abundant silver ions during the first week and then continuously and gradually release them until week 26. Taking into account that Ag_3_PO_4_-modified CAp HC scaffolds (Ag860-HC) achieved anti-infective bone regeneration, the silver-ion-releasing behavior is considered suitable for preventing bacterial adhesion to the scaffold during and immediately after surgery and achieving subsequent bone replacement, while maintaining antibacterial activity, without complications from prolonged exposure to silver ions. Furthermore, our modification method could easily control the silver concentration in the HC scaffold by controlling the concentration of the AgNO_3_ solution. A key factor affecting the silver concentration is the pH of the AgNO_3_ solution, which varies significantly around a pH of 6. Guidry et al. reported that the solubility of CAp dramatically increases as the pH of the solution decreases below 6 [77]. Proving this report, we observed that CAp easily dissolved in AgNO_3_ solutions with pH < 6, which promoted the subsequent precipitation of Ag_3_PO_4_. Conversely, in AgNO_3_ solutions (≤5 mmol/L) with pH ≥ 6, the silver concentration linearly and gradually increased with increasing AgNO_3_ concentration. Thus, mild dissolution-precipitation reactions occur in AgNO_3_ solutions with a concentration <5 mmol/L, which is favorable for controlling the silver concentration.

In terms of overall in vivo anti-infectivity, bone regeneration, scaffold resorption, pigmentation, and toxicity, the optimal silver concentration range for Ag_3_PO_4_-modified CAp HC scaffolds is discussed below. The minimum silver concentration for Ag_3_PO_4_-modified CAp HC scaffolds that can both prevent infection and bone formation in the presence of bacteria is 864 ± 179 ppm (Ag860-HC). In the absence of bacteria, no significant differences in scaffold resorption and bone formation were detected between HC scaffolds with and without in vitro cytotoxicity. Conversely, with respect to bone formation, Ag5200-HC with a silver content of 5200 ± 471 ppm—at which in vitro cytotoxicity was observed—showed greater bone formation at week 4 than scaffolds with other silver concentrations. Previous in vitro studies have demonstrated that silver ions enhance the proliferation and differentiation of mesenchymal stem cells and osteoblast-like cells by activating the signaling pathways of integrin α5, transforming growth factor beta, and bone morphogenic proteins as well as upregulating osteogenesis-related genes, zinc finger-containing transcription factor osterix, and extracellular matrix mineralization [[21], [22], [23], [24], [25]]. Thus, the results of our present study are consistent with those of the previous studies in terms of the fact that silver phosphate modification promotes bone formation. However, the effective silver concentration differs between our in vivo and previous in vitro studies. Previous in vitro studies demonstrated that 431 and 250 ng/mL of silver enhanced the proliferation and osteogenic differentiation of mesenchymal stem cells, respectively [[78], [79], [80]]. Furthermore, the in vitro differentiation of MC3T3 cells was enhanced when the accumulated concentration of silver ions released from the scaffold was 55.4 ng/mL on day 14 and 58.1 ng/mL on day 21 [[79], [80], [81]]. These reported silver concentrations were 10,000–100000 times lower than those promoting in vivo bone formation in our present study. Thus, to obtain an osteogenesis-promoting effect in vivo, considerably higher concentrations of silver are required than those effective in vitro. This is may be because the in vivo environment is a dynamic system and differs from in vitro closed systems; consequently, the silver ions released from the scaffold are easily diffused. Furthermore, even Ag21000-HC with a silver concentration of 21,000 ± 433 ppm that exhibited in vitro cytotoxicity showed no in vivo acute systemic toxicity. Thus, even silver concentrations approximately nine times higher than those at which in vitro cytotoxicity occurs are tolerated in vivo. This finding coincides with the finding reported by Yuan et al. that β-tricalcium phosphate (β-TCP) scaffolds containing 50,000 and 100,000 ppm of silver showed no toxicity in vivo and formed almost the same amount of new bone as pure β-TCP scaffolds [61]. These findings indicate that silver does not inhibit bone formation when silver concentration is increased to 100,000 ppm, which is may be attributed to the diffusion of silver ions in vivo. However, our present study demonstrated that pigmentation occurred and silver-phagocytized cells appeared in the HC scaffolds with silver concentrations above 5200 ± 471 ppm (Ag5200-HC and Ag21000-HC). Based on the above findings and from a comprehensive understanding of in vivo anti-infectivity, bone regeneration, scaffold resorption, pigmentation, and toxicity, we concluded that the optimal silver concentration range for Ag_3_PO_4_-modified CAp HC scaffolds was 285–864 ppm.

Various types of silver-containing osteoconductive inorganics, such as hydroxyapatite (HAp) [27,28,32,33,39], β-TCP [29,38,43,44], biphasic calcium phosphate (BCP) consisting of HAp and β-TCP [30,35,41,63], amorphous calcium phosphate (ACP) [44], strontium phosphate silicate apatite [31], bioactive glasses [34,36,37,43], have been reported. In most of these studies using calcium phosphates, silver ions were doped into calcium phosphates by substituting silver ions for calcium ions [[27], [28], [29], [30], [31], [32],^38^,^39^,^41^,^44^]. However, the performance of these materials has been evaluated only in vitro, and their effectiveness in vivo has not been demonstrated. Nevertheless, the studies provide in vitro antibacterial characteristics of silver-doped calcium phosphates. Although 10 mol% silver-doped HAp showed antibacterial activity [27], 1 mol% silver-doped HAp did not [28]. Both 2.87 and 5.75 mol% silver-doped β-TCP exhibited antibacterial activity [29]. No difference in antibacterial activity between pure BCP and 4 mol% silver-doped BCP was observed, indicating that 4 mol% silver-doped BCP showed no antibacterial activity [30]. These findings suggest that the relationship between antibacterial activity and silver concentration depends on the solubility of the base material, i.e., HAp, β-TCP, and BCP. The release rate of silver ions from the base material is relatively low, which seems suitable to prevent delayed infection. However, to prevent surgical site infection that occurs in the early postoperative period, large amounts of silver ions appear appropriate to be released immediately after implantation to destroy almost all the bacteria. In fact, the slow release rate of silver ions from silver-doped HAp (0.4 and 2.9 ppm at 15 and 45 days, respectively) resulted in residual bacteria (67 %, 17 %, 1 %, and 9 % of *E. coli* and 43 %, 23 %, 19 %, and 22 % of *S. aureus* remained after 6, 12, 24, and 48 h of incubation) [27]. Thus, the two-stage release of silver ions, that is, the combined release of large amounts early after implantation and the subsequent gradual release, is considered suitable for preventing both surgical site and delayed infections. The modification approach used in our present study with the two-stage release of silver ions may have enabled the Ag_3_PO_4_-modified CAp HC scaffolds to achieve anti-infective bone tissue engineering capabilities.

Furthermore, this study showed that CAp HC scaffolds without Ag_3_PO_4_ modification cause lower rates of osteonecrosis and osteomyelitis than clinically used 3D porous scaffolds. We speculate that this is because the HC structure allows more blood to flow into the scaffold than the 3D porous structure. Therefore, bacteria are washed away from the scaffold and are less likely to concentrate within the scaffold. In fact, we observed that blood permeates into the HC scaffolds immediately after their implantation in the bone defect, whereas blood requires longer duration to permeate the 3D porous scaffolds. The new finding in this study suggests that infection prevention can be achieved by controlling the scaffold structure. The scaffold structure has been known to be a key factor for bone regeneration, and uniaxial channel structures, such as HC and tubular structures, have been used to promote vascular and bone regeneration [81,82]. Hence, the HC structure is competent for achieving bone and blood vessel regeneration while preventing infection.

To date, no CAp-block scaffolds have been approved for clinical use. Therefore, the 3D porous scaffolds used in this study are composed of β-TCP, a typical composition of bioresorbable calcium phosphate scaffolds. Considering only the composition, β-TCP may be less likely to cause infection than CAp because β-TCP is resorbed faster than CAp. Thus, although CAp HC scaffolds were expected to be compositionally more prone to infection than the clinically used 3D porous scaffolds, the results were the opposite, highlighting the anti-infective properties of the HC structure. However, the composition of the scaffolds should be identical to accurately elucidate the effects of the scaffold structure. Therefore, we aim to elucidate the effect of structure on the anti-infective properties by comparing scaffolds with the same composition but different structures.

## 5 Conclusion

In this study, to determine the optimal silver contents for pigmentation-free anti-infective bone regeneration, CAp HC scaffolds with 11 different concentrations of Ag_3_PO_4_ (0–21000 ppm) were fabricated. Ag_3_PO_4_-modified CAp HC scaffolds with silver contents of 864 ppm regenerated bone while preventing abscess formation, osteonecrosis, and osteomyelitis. Interestingly, even CAp HC scaffolds without Ag_3_PO_4_ modification had a lower incidence of osteonecrosis and osteomyelitis than clinically used 3D porous scaffolds. Furthermore, increasing the silver content to 21,000 ppm did not adversely affect bone formation or scaffold resorption or cause acute systemic toxicity, although silver content at ≥ 5200 ppm showed in vitro cytotoxicity. However, pigmentation occurred at that silver content (5200 ppm), whereas no pigmentation occurred for silver concentrations ≤2300 ppm. Based on the above results, we concluded that the optimal silver concentration range was 864–2300 ppm, allowing bone regeneration without infection or pigmentation. The achievement of pigmentation-free anti-infective bone regeneration was attributed to the surface modification method, which resulted in an abundant release of silver ions immediately after implantation to completely kill bacteria, followed by a gradual release over several months to prevent bacterial growth. Another reason may be that the scaffold structure prevented an increase in the concentration of bacteria within the scaffold.

## Funding

10.13039/100009619Japan Agency for Medical Research and Development, JP24ym0126098h0003 (KH).

10.13039/100009619Japan Agency for Medical Research and Development, JP24ym0126811j0003 (KH).

10.13039/501100001691Japan Society for the Promotion of Science, JP23K18593 (KH)

10.13039/501100001691Japan Society for the Promotion of Science, JP22H03954 (KH)

## Data and materials availability

Data available on request from the authors.

## CRediT authorship contribution statement

**Koichiro Hayashi:** Writing – review & editing, Writing – original draft, Visualization, Supervision, Resources, Project administration, Methodology, Investigation, Data curation, Conceptualization. **Masaya Shimabukuro:** Writing – review & editing, Investigation. **Cheng Zhang:** Investigation. **Ahmad Nazir Taleb Alashkar:** Investigation. **Ryo Kishida:** Writing – review & editing, Investigation. **Akira Tsuchiya:** Writing – review & editing, Investigation. **Kunio Ishikawa:** Validation, Project administration.

## Declaration of competing interest

The authors declare the following financial interests/personal relationships which may be considered as potential competing interests:

Koichiro Hayashi reports financial support was provided by Japan Society for the Promotion of Science. Koichiro Hayashi reports financial support was provided by Japan Agency for Medical Research and Development. If there are other authors, they declare that they have no known competing financial interests or personal relationships that could have appeared to influence the work reported in this paper.

## Data Availability

Data will be made available on request.
