# Supplementary material for: Silver phosphate-modified carbonate apatite honeycomb scaffolds for anti-infective and pigmentation-free bone tissue engineering
Source: Mater Today Bio. 2024 Jul 18;27:101161. doi: 10.1016/j.mtbio.2024.101161 (PMC11326936; doi:10.1016/j.mtbio.2024.101161)
Supplement: Multimedia component 1 [file mmc1.pdf]

Supplementary Materials

Silver phosphate-modified carbonate apatite honeycomb scaffolds for anti-infective and pigmentation-free bone tissue engineering

Koichiro Hayashi,[a*] Masaya Shimabukuro,[a,b] Cheng Zhang,[a] Ahmad Nazir Taleb Alashkar,[a] Ryo Kishida,[a] Akira Tsuchiya,[a] and Kunio Ishikawa[a]

Affiliations:

[a] Department of Biomaterials, Faculty of Dental Science, Kyushu University 3-1-1 Maidashi, Higashi-ku, Fukuoka 812-8582, Japan

[b] Institute of Biomaterials and Bioengineering, Tokyo Medical and Dental University, 2-3-10, Kanda-Surugadai, Chiyoda-ku, Tokyo 101-0062, Japan

***Corresponding author:** Koichiro Hayashi

e-mail: khayashi@dent.kyushu-u.ac.jp

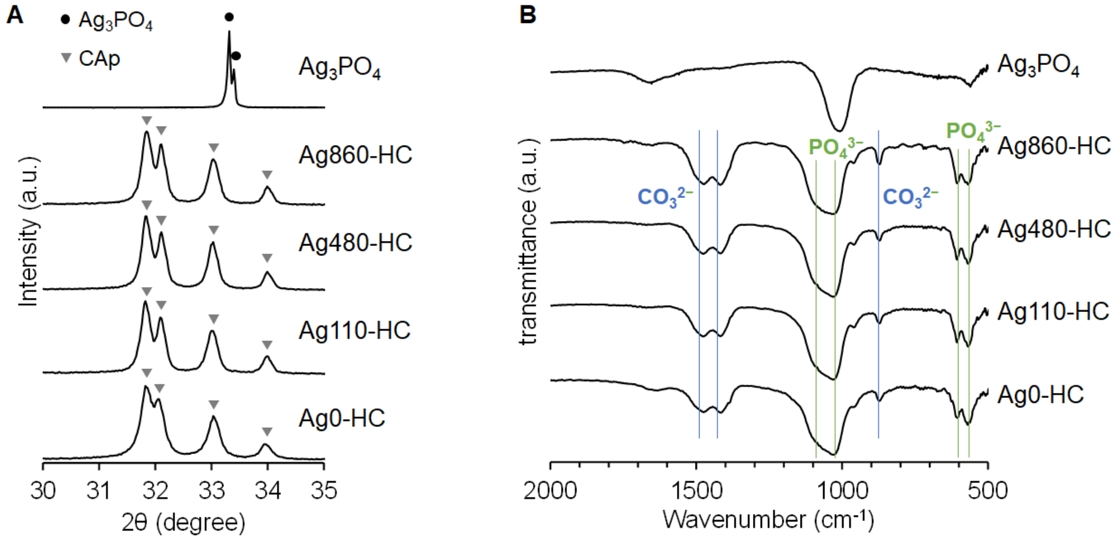

Fig. S1. Structural characterization results of carbonate apatite (CAp) honeycomb (HC) scaffolds. (A) X-ray diffraction patterns and (B) Fourier transform infrared spectra of Ag0-HC, Ag110-HC, Ag480-HC, and Ag860-HC.

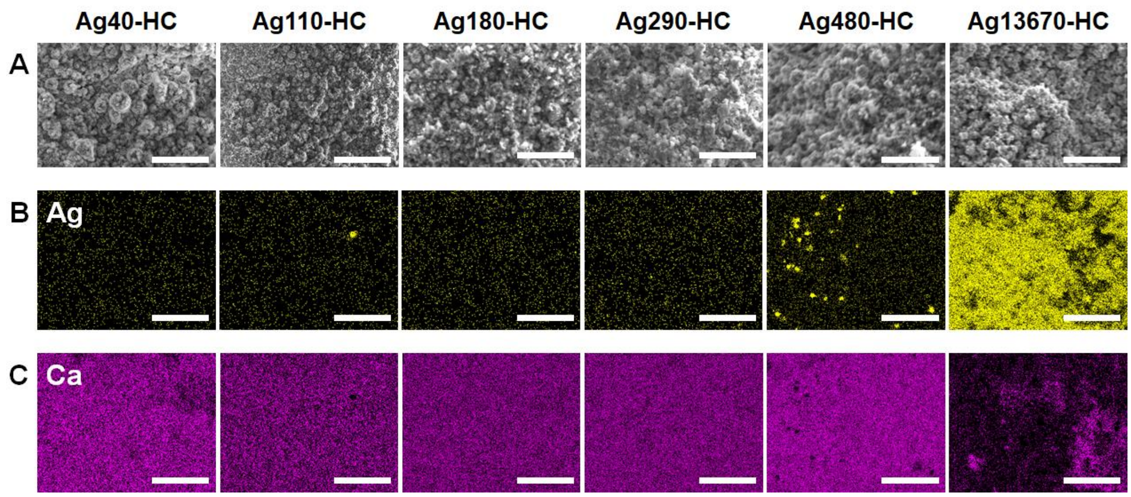

Fig. S2. (**A**) Scanning electron microscopy images and energy dispersive X-ray maps of (**B**) Ag and (**C**) Ca of Ag40-HC, Ag110-HC, Ag180-HC, Ag290-HC, Ag480-HC, and Ag13670-HC. Scale bar: 20 μm.

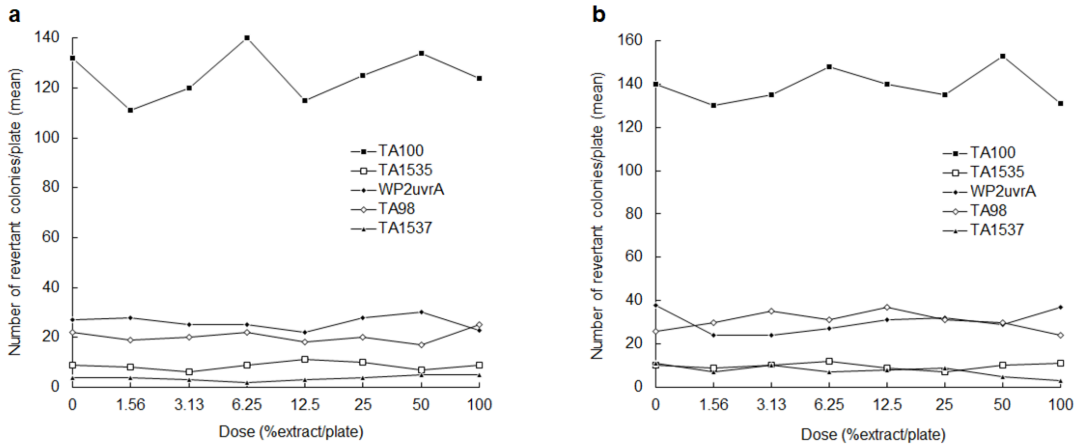

Fig. S3. Results of bacterial reverse mutation test (a) without and (b) with metabolic activation.

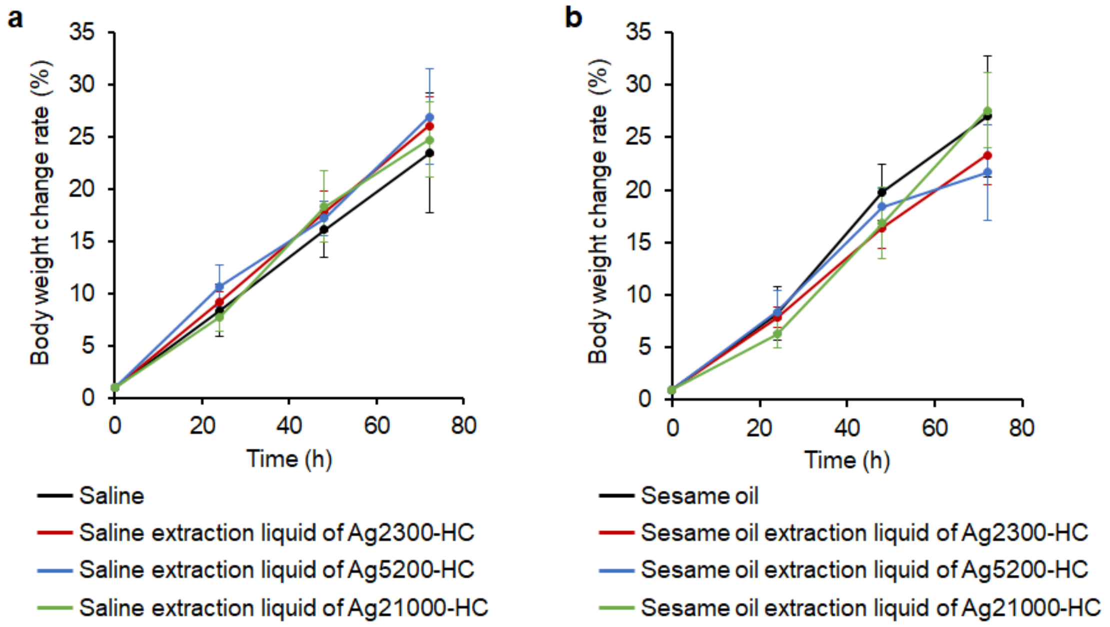

Fig. S4. Body weight increase rates at 24, 48, and 72 h after (a) intravenous injection of saline and saline extraction liquids of Ag2300-HC, Ag5200-HC, and Ag21000-HC and (b) intraperitoneal injection of sesame oil and sesame oil extraction liquid Ag2300-HC, Ag5200-HC, and Ag21000-HC.

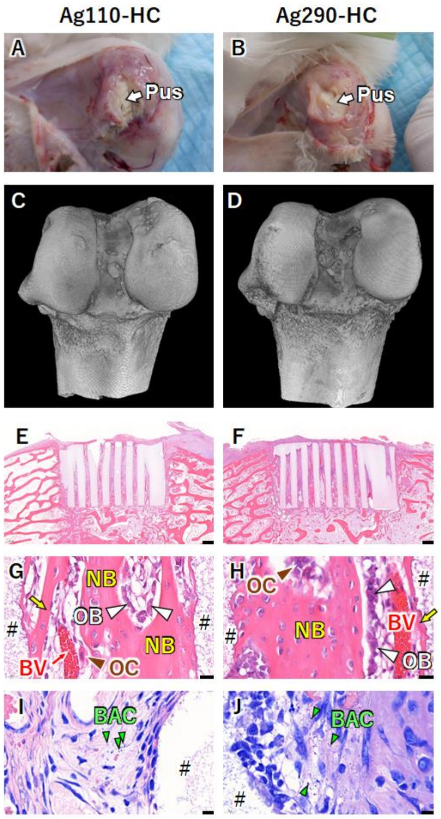

Fig. S5. In vivo results on anti-infective bone regeneration at two weeks after implantation. Images of femoral regions with skin incision in (**A**) Ag110-HC- and (**B**) Ag290-HC-implanted groups. Microcomputed tomography (μ-CT) images of rabbit femurs in (**C**) Ag110-HC- and (**D**) Ag290-HC-implanted groups. Hematoxylin and eosin (HE)-stained histological sections of (**E**) Ag110-HC- and (**F**) Ag290-HC-implanted groups. (**G, H**) High-magnification HE-stained images. Giemsa-stained histological sections of (**I**) Ag110-HC- and (**J**) Ag290-HC-implanted groups. Scale bars: (**E, F**) 500 μm, (**G, H**) 20 μm, and (**I, J**) 10 μm. NB and yellow arrows indicate new bones. BV and red arrows indicate blood vessels. OB and white arrowheads indicate osteoblasts. OC and blown arrowheads indicate osteoclasts. # and BAC indicate the remaining material and bacteria, respectively.

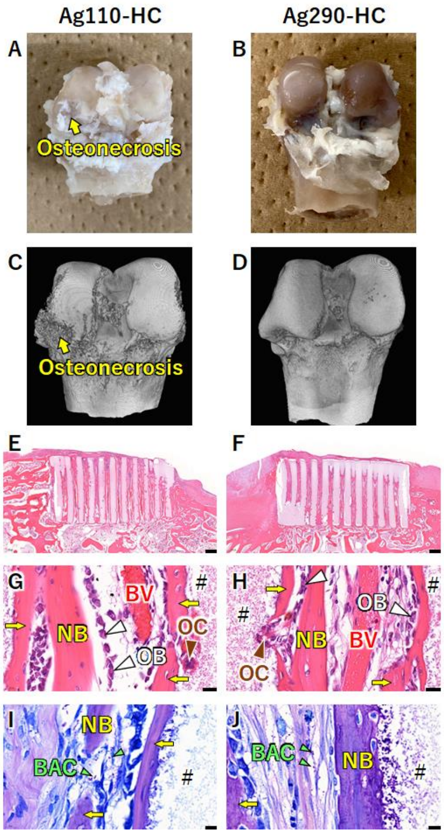

Fig. S6. In vivo results on anti-infective bone regeneration at four weeks after implantation. Images of femoral regions with skin incision in (**A**) Ag110-HC- and (**B**) Ag290-HC-implanted groups. μ-CT images of rabbit femurs in (**C**) Ag110-HC- and (**D**) Ag290-HC-implanted groups. HE-stained histological sections of (**E**) Ag110-HC- and (**F**) Ag290-HC-implanted groups. (**G, H**) High-magnification HE-stained images. Giemsa-stained histological sections of (**I**) Ag110-HC- and (**J**) Ag290-HC-implanted groups. Scale bars: (**E, F**) 500 μm, (**G, H**) 20 μm, and (**I, J**) 10 μm. NB and yellow arrows indicate new bones. BV and red arrows indicate blood vessels. OB and white arrowheads indicate osteoblasts. OC and blown arrowheads indicate osteoclasts. # and BAC indicate the remaining material and bacteria, respectively.

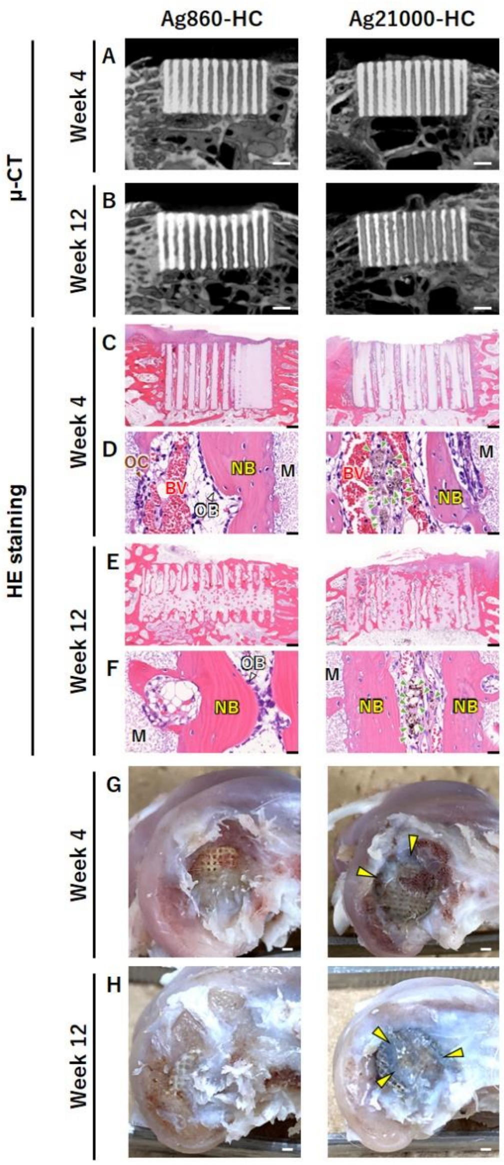

Fig. S7. Analysis of in vivo bone regeneration and pigmentation for different scaffolds at weeks 4 and 12. μ-CT images of Ag860-HC and Ag21000-HC scaffolds at weeks (**A**) 4 and (**B**) week 12 after implantation. HE staining of Ag860-HC and Ag21000-HC scaffolds at weeks (**C**) 4 and (**D**) 12 after implantation. #, Empty, NB, BV, OB, and OC indicate the remaining material, empty pores, new bone, blood vessels, osteoblasts, and osteoclasts, respectively. Green arrowheads indicate brown-stained giant cells, which are considered macrophages that phagocytize silver. Gross anatomical findings at weeks

(**G**) 4 and (**H**) 12. Yellow arrowheads indicate pigmented regions. Scale bars: (A, **B**) 1 mm, (**C**, **E**) 500 µm, (**D**, **F**) 20 µm, and (**G**, **H**) 1 mm.

Table S1. Concentrations of positive control materials.

Bacteria	In the absence of metabolic activation		In the presence of metabolic activating	
	Positive control	Concentration (μg/plate)	Positive control	Concentration (μg/plate)
TA100	4NQO	0.1	2AA	1
TA1535	NaN_3	0.5	2AA	2
WP2*uvrA*	4NQO	0.2	2AA	10
TA98	4NQO	0.2	2AA	0.5
TA1537	9AA	80	2AA	2

Table S2. Evaluation of acute systemic toxicity. i.v. and i.p. indicate intravenous and intraperitoneal injections, respectively.

Group	Test solution	Administration	Animal number
1	Saline (control)	i.v.	5
2	Saline extraction liquid of Ag2300-HC	i.v.	5
3	Saline extraction liquid of Ag5200-HC	i.v.	5
4	Saline extraction liquid of Ag21000-HC	i.v.	5
5	Sesame oil (control)	i.p.	5
6	Sesame oil extraction liquid of Ag2300-HC	i.p.	5
7	Sesame oil extraction liquid of Ag5200-HC	i.p.	5
8	Sesame oil extraction liquid of Ag21000-HC	i.p.	5

Table S3. List of observations of general conditions

Observation item	Symptom
Appearance	Death, debilitation
Body posture and behavior	Stooping, decreased spontaneous movements, gait abnormalities
Nervous system	Tremor, spasm, hypotonia
Breathing	Breathlessness, dyspnea, apnea, hyperpnea
Hair	Standing hair, dirt in the hair
Oculus	Bleeding, protruding eyeballs, droopy eyelids, conjunctival swelling
Ear, nose, mouth	Ear discharge, nasal discharge, drooling
Egestion	Polyuria, soft stools, bad odor
Other	Symptoms that are not listed above

Table S4. Results of multiple comparisons using the Tukey–Kramer test for the data shown in Figure 5C.

Comparison	*p*
Ag290-HC 3% vs. Ag5200-HC 100%	< .0001
Ag290-HC 3% vs. Ag2300-HC 100%	< .0001
Ag290-HC 3% vs. Ag180-HC 100%	< .0001
Ag290-HC 3% vs. Ag480-HC 100%	< .0001
Ag290-HC 3% vs. Ag480-HC 30%	< .0001
Ag290-HC 3% vs. Ag480-HC 50%	< .0001
Ag290-HC 3% vs. Ag860-HC 100%	< .0001
Ag290-HC 3% vs. Ag290-HC 100%	< .0001
Ag290-HC 3% vs. Ag180-HC 30%	< .0001
Ag290-HC 3% vs. Ag480-HC 10%	< .0001
Ag290-HC 3% vs. Ag180-HC 10%	< .0001
Ag290-HC 3% vs. Ag180-HC 50%	< .0001
Ag290-HC 3% vs. Ag860-HC 30%	< .0001
Ag290-HC 3% vs. Ag2300-HC 10%	< .0001
Ag290-HC 3% vs. Ag180-HC 3%	< .0001
Ag290-HC 3% vs. Ag5200-HC 50%	< .0001
Ag290-HC 3% vs. Ag2300-HC 3%	< .0001
Ag290-HC 3% vs. Ag2300-HC 30%	< .0001
Ag290-HC 3% vs. Ag2300-HC 50%	< .0001
Ag290-HC 3% vs. Ag290-HC 30%	< .0001
Ag290-HC 3% vs. Ag860-HC 10%	0.0001

Ag290-HC 3% vs. Ag290-HC 50%	0.0003
Ag290-HC 3% vs. Ag5200-HC 30%	0.0042
Ag290-HC 3% vs. Ag480-HC 3%	0.0055
Ag290-HC 3% vs. Ag860-HC 50%	0.0401
Ag290-HC 3% vs. Ag0-HC 50%	0.1949
Ag290-HC 3% vs. Ag0-HC 100%	0.2482
Ag290-HC 3% vs. Ag290-HC 10%	0.7337
Ag290-HC 3% vs. Ag0-HC 30%	0.789
Ag290-HC 3% vs. Ag0-HC 10%	0.8095
Ag290-HC 3% vs. Ag5200-HC 10%	0.8351
Ag290-HC 3% vs. Ag860-HC 3%	0.9913
Ag290-HC 3% vs. Ag5200-HC 3%	1
Ag290-HC 3% vs. Ag0-HC 3%	1
Ag0-HC 3% vs. Ag5200-HC 100%	< .0001
Ag0-HC 3% vs. Ag2300-HC 100%	< .0001
Ag0-HC 3% vs. Ag180-HC 100%	< .0001
Ag0-HC 3% vs. Ag480-HC 100%	< .0001
Ag0-HC 3% vs. Ag480-HC 30%	< .0001
Ag0-HC 3% vs. Ag480-HC 50%	< .0001
Ag0-HC 3% vs. Ag860-HC 100%	< .0001
Ag0-HC 3% vs. Ag290-HC 100%	< .0001
Ag0-HC 3% vs. Ag180-HC 30%	< .0001
Ag0-HC 3% vs. Ag480-HC 10%	< .0001
Ag0-HC 3% vs. Ag180-HC 10%	< .0001

Ag0-HC 3% vs. Ag180-HC 50%	< .0001
Ag0-HC 3% vs. Ag860-HC 30%	< .0001
Ag0-HC 3% vs. Ag2300-HC 10%	0.0001
Ag0-HC 3% vs. Ag180-HC 3%	0.0003
Ag0-HC 3% vs. Ag5200-HC 50%	0.0004
Ag0-HC 3% vs. Ag2300-HC 3%	0.0005
Ag0-HC 3% vs. Ag2300-HC 30%	0.0023
Ag0-HC 3% vs. Ag2300-HC 50%	0.0028
Ag0-HC 3% vs. Ag290-HC 30%	0.0063
Ag0-HC 3% vs. Ag860-HC 10%	0.0153
Ag0-HC 3% vs. Ag290-HC 50%	0.0261
Ag0-HC 3% vs. Ag5200-HC 30%	0.1848
Ag0-HC 3% vs. Ag480-HC 3%	0.219
Ag0-HC 3% vs. Ag860-HC 50%	0.6105
Ag0-HC 3% vs. Ag0-HC 50%	0.9371
Ag0-HC 3% vs. Ag0-HC 100%	0.9635
Ag0-HC 3% vs. Ag290-HC 10%	0.9999
Ag0-HC 3% vs. Ag0-HC 30%	1
Ag0-HC 3% vs. Ag0-HC 10%	1
Ag0-HC 3% vs. Ag5200-HC 10%	1
Ag0-HC 3% vs. Ag860-HC 3%	1
Ag0-HC 3% vs. Ag5200-HC 3%	1
Ag5200-HC 3% vs. Ag5200-HC 100%	< .0001
Ag5200-HC 3% vs. Ag2300-HC 100%	< .0001

Ag5200-HC 3% vs. Ag180-HC 100%	< .0001
Ag5200-HC 3% vs. Ag480-HC 100%	< .0001
Ag5200-HC 3% vs. Ag480-HC 30%	< .0001
Ag5200-HC 3% vs. Ag480-HC 50%	< .0001
Ag5200-HC 3% vs. Ag860-HC 100%	< .0001
Ag5200-HC 3% vs. Ag290-HC 100%	< .0001
Ag5200-HC 3% vs. Ag180-HC 30%	< .0001
Ag5200-HC 3% vs. Ag480-HC 10%	< .0001
Ag5200-HC 3% vs. Ag180-HC 10%	< .0001
Ag5200-HC 3% vs. Ag180-HC 50%	< .0001
Ag5200-HC 3% vs. Ag860-HC 30%	0.0002
Ag5200-HC 3% vs. Ag2300-HC 10%	0.0002
Ag5200-HC 3% vs. Ag180-HC 3%	0.0005
Ag5200-HC 3% vs. Ag5200-HC 50%	0.0007
Ag5200-HC 3% vs. Ag2300-HC 3%	0.0008
Ag5200-HC 3% vs. Ag2300-HC 30%	0.0039
Ag5200-HC 3% vs. Ag2300-HC 50%	0.0047
Ag5200-HC 3% vs. Ag290-HC 30%	0.0106
Ag5200-HC 3% vs. Ag860-HC 10%	0.0246
Ag5200-HC 3% vs. Ag290-HC 50%	0.0409
Ag5200-HC 3% vs. Ag5200-HC 30%	0.2543
Ag5200-HC 3% vs. Ag480-HC 3%	0.2964
Ag5200-HC 3% vs. Ag860-HC 50%	0.7142
Ag5200-HC 3% vs. Ag0-HC 50%	0.9698

Ag5200-HC 3% vs. Ag0-HC 100%	0.9843
Ag5200-HC 3% vs. Ag290-HC 10%	1
Ag5200-HC 3% vs. Ag0-HC 30%	1
Ag5200-HC 3% vs. Ag0-HC 10%	1
Ag5200-HC 3% vs. Ag5200-HC 10%	1
Ag5200-HC 3% vs. Ag860-HC 3%	1
Ag860-HC 3% vs. Ag5200-HC 100%	< .0001
Ag860-HC 3% vs. Ag2300-HC 100%	< .0001
Ag860-HC 3% vs. Ag180-HC 100%	< .0001
Ag860-HC 3% vs. Ag480-HC 100%	< .0001
Ag860-HC 3% vs. Ag480-HC 30%	< .0001
Ag860-HC 3% vs. Ag480-HC 50%	< .0001
Ag860-HC 3% vs. Ag860-HC 100%	< .0001
Ag860-HC 3% vs. Ag290-HC 100%	< .0001
Ag860-HC 3% vs. Ag180-HC 30%	< .0001
Ag860-HC 3% vs. Ag480-HC 10%	< .0001
Ag860-HC 3% vs. Ag180-HC 10%	0.0002
Ag860-HC 3% vs. Ag180-HC 50%	0.0006
Ag860-HC 3% vs. Ag860-HC 30%	0.003
Ag860-HC 3% vs. Ag2300-HC 10%	0.0038
Ag860-HC 3% vs. Ag180-HC 3%	0.0088
Ag860-HC 3% vs. Ag5200-HC 50%	0.0114
Ag860-HC 3% vs. Ag2300-HC 3%	0.0129
Ag860-HC 3% vs. Ag2300-HC 30%	0.0462

Ag860-HC 3% vs. Ag2300-HC 50%	0.0538
Ag860-HC 3% vs. Ag290-HC 30%	0.101
Ag860-HC 3% vs. Ag860-HC 10%	0.1898
Ag860-HC 3% vs. Ag290-HC 50%	0.27
Ag860-HC 3% vs. Ag5200-HC 30%	0.7565
Ag860-HC 3% vs. Ag480-HC 3%	0.8028
Ag860-HC 3% vs. Ag860-HC 50%	0.9875
Ag860-HC 3% vs. Ag0-HC 50%	1
Ag860-HC 3% vs. Ag0-HC 100%	1
Ag860-HC 3% vs. Ag290-HC 10%	1
Ag860-HC 3% vs. Ag0-HC 30%	1
Ag860-HC 3% vs. Ag0-HC 10%	1
Ag860-HC 3% vs. Ag5200-HC 10%	1
Ag5200-HC 10% vs. Ag5200-HC 100%	< .0001
Ag5200-HC 10% vs. Ag2300-HC 100%	< .0001
Ag5200-HC 10% vs. Ag180-HC 100%	< .0001
Ag5200-HC 10% vs. Ag480-HC 100%	< .0001
Ag5200-HC 10% vs. Ag480-HC 30%	< .0001
Ag5200-HC 10% vs. Ag480-HC 50%	< .0001
Ag5200-HC 10% vs. Ag860-HC 100%	< .0001
Ag5200-HC 10% vs. Ag290-HC 100%	< .0001
Ag5200-HC 10% vs. Ag180-HC 30%	0.0001
Ag5200-HC 10% vs. Ag480-HC 10%	0.0003
Ag5200-HC 10% vs. Ag180-HC 10%	0.0024

Ag5200-HC 10% vs. Ag180-HC 50%	0.0062
Ag5200-HC 10% vs. Ag860-HC 30%	0.0233
Ag5200-HC 10% vs. Ag2300-HC 10%	0.0291
Ag5200-HC 10% vs. Ag180-HC 3%	0.0586
Ag5200-HC 10% vs. Ag5200-HC 50%	0.0725
Ag5200-HC 10% vs. Ag2300-HC 3%	0.0799
Ag5200-HC 10% vs. Ag2300-HC 30%	0.2162
Ag5200-HC 10% vs. Ag2300-HC 50%	0.2421
Ag5200-HC 10% vs. Ag290-HC 30%	0.3763
Ag5200-HC 10% vs. Ag860-HC 10%	0.5589
Ag5200-HC 10% vs. Ag290-HC 50%	0.6778
Ag5200-HC 10% vs. Ag5200-HC 30%	0.9791
Ag5200-HC 10% vs. Ag480-HC 3%	0.987
Ag5200-HC 10% vs. Ag860-HC 50%	1
Ag5200-HC 10% vs. Ag0-HC 50%	1
Ag5200-HC 10% vs. Ag0-HC 100%	1
Ag5200-HC 10% vs. Ag290-HC 10%	1
Ag5200-HC 10% vs. Ag0-HC 30%	1
Ag5200-HC 10% vs. Ag0-HC 10%	1
Ag0-HC 10% vs. Ag5200-HC 100%	< .0001
Ag0-HC 10% vs. Ag2300-HC 100%	< .0001
Ag0-HC 10% vs. Ag180-HC 100%	< .0001
Ag0-HC 10% vs. Ag480-HC 100%	< .0001
Ag0-HC 10% vs. Ag480-HC 30%	< .0001

Ag0-HC 10% vs. Ag480-HC 50%	< .0001
Ag0-HC 10% vs. Ag860-HC 100%	< .0001
Ag0-HC 10% vs. Ag290-HC 100%	< .0001
Ag0-HC 10% vs. Ag180-HC 30%	0.0002
Ag0-HC 10% vs. Ag480-HC 10%	0.0003
Ag0-HC 10% vs. Ag180-HC 10%	0.0028
Ag0-HC 10% vs. Ag180-HC 50%	0.0073
Ag0-HC 10% vs. Ag860-HC 30%	0.027
Ag0-HC 10% vs. Ag2300-HC 10%	0.0336
Ag0-HC 10% vs. Ag180-HC 3%	0.0669
Ag0-HC 10% vs. Ag5200-HC 50%	0.0825
Ag0-HC 10% vs. Ag2300-HC 3%	0.0907
Ag0-HC 10% vs. Ag2300-HC 30%	0.2392
Ag0-HC 10% vs. Ag2300-HC 50%	0.2668
Ag0-HC 10% vs. Ag290-HC 30%	0.4077
Ag0-HC 10% vs. Ag860-HC 10%	0.5934
Ag0-HC 10% vs. Ag290-HC 50%	0.7102
Ag0-HC 10% vs. Ag5200-HC 30%	0.9843
Ag0-HC 10% vs. Ag480-HC 3%	0.9905
Ag0-HC 10% vs. Ag860-HC 50%	1
Ag0-HC 10% vs. Ag0-HC 50%	1
Ag0-HC 10% vs. Ag0-HC 100%	1
Ag0-HC 10% vs. Ag290-HC 10%	1
Ag0-HC 10% vs. Ag0-HC 30%	1

Ag0-HC 30% vs. Ag5200-HC 100%	< .0001
Ag0-HC 30% vs. Ag2300-HC 100%	< .0001
Ag0-HC 30% vs. Ag180-HC 100%	< .0001
Ag0-HC 30% vs. Ag480-HC 100%	< .0001
Ag0-HC 30% vs. Ag480-HC 30%	< .0001
Ag0-HC 30% vs. Ag480-HC 50%	< .0001
Ag0-HC 30% vs. Ag860-HC 100%	< .0001
Ag0-HC 30% vs. Ag290-HC 100%	< .0001
Ag0-HC 30% vs. Ag180-HC 30%	0.0002
Ag0-HC 30% vs. Ag480-HC 10%	0.0004
Ag0-HC 30% vs. Ag180-HC 10%	0.0032
Ag0-HC 30% vs. Ag180-HC 50%	0.0083
Ag0-HC 30% vs. Ag860-HC 30%	0.0302
Ag0-HC 30% vs. Ag2300-HC 10%	0.0374
Ag0-HC 30% vs. Ag180-HC 3%	0.0737
Ag0-HC 30% vs. Ag5200-HC 50%	0.0907
Ag0-HC 30% vs. Ag2300-HC 3%	0.0995
Ag0-HC 30% vs. Ag2300-HC 30%	0.2574
Ag0-HC 30% vs. Ag2300-HC 50%	0.2863
Ag0-HC 30% vs. Ag290-HC 30%	0.432
Ag0-HC 30% vs. Ag860-HC 10%	0.6191
Ag0-HC 30% vs. Ag290-HC 50%	0.7337
Ag0-HC 30% vs. Ag5200-HC 30%	0.9875
Ag0-HC 30% vs. Ag480-HC 3%	0.9926

Ag0-HC 30% vs. Ag860-HC 50%	1
Ag0-HC 30% vs. Ag0-HC 50%	1
Ag0-HC 30% vs. Ag0-HC 100%	1
Ag0-HC 30% vs. Ag290-HC 10%	1
Ag290-HC 10% vs. Ag5200-HC 100%	< .0001
Ag290-HC 10% vs. Ag2300-HC 100%	< .0001
Ag290-HC 10% vs. Ag180-HC 100%	< .0001
Ag290-HC 10% vs. Ag480-HC 100%	< .0001
Ag290-HC 10% vs. Ag480-HC 30%	< .0001
Ag290-HC 10% vs. Ag480-HC 50%	< .0001
Ag290-HC 10% vs. Ag860-HC 100%	< .0001
Ag290-HC 10% vs. Ag290-HC 100%	< .0001
Ag290-HC 10% vs. Ag180-HC 30%	0.0003
Ag290-HC 10% vs. Ag480-HC 10%	0.0005
Ag290-HC 10% vs. Ag180-HC 10%	0.0044
Ag290-HC 10% vs. Ag180-HC 50%	0.0112
Ag290-HC 10% vs. Ag860-HC 30%	0.0394
Ag290-HC 10% vs. Ag2300-HC 10%	0.0486
Ag290-HC 10% vs. Ag180-HC 3%	0.0935
Ag290-HC 10% vs. Ag5200-HC 50%	0.114
Ag290-HC 10% vs. Ag2300-HC 3%	0.1247
Ag290-HC 10% vs. Ag2300-HC 30%	0.3067
Ag290-HC 10% vs. Ag2300-HC 50%	0.3387
Ag290-HC 10% vs. Ag290-HC 30%	0.4946

Ag290-HC 10% vs. Ag860-HC 10%	0.6819
Ag290-HC 10% vs. Ag290-HC 50%	0.789
Ag290-HC 10% vs. Ag5200-HC 30%	0.9932
Ag290-HC 10% vs. Ag480-HC 3%	0.9963
Ag290-HC 10% vs. Ag860-HC 50%	1
Ag290-HC 10% vs. Ag0-HC 50%	1
Ag290-HC 10% vs. Ag0-HC 100%	1
Ag0-HC 100% vs. Ag5200-HC 100%	< .0001
Ag0-HC 100% vs. Ag2300-HC 100%	< .0001
Ag0-HC 100% vs. Ag180-HC 100%	< .0001
Ag0-HC 100% vs. Ag480-HC 100%	< .0001
Ag0-HC 100% vs. Ag480-HC 30%	0.0003
Ag0-HC 100% vs. Ag480-HC 50%	0.0004
Ag0-HC 100% vs. Ag860-HC 100%	0.0008
Ag0-HC 100% vs. Ag290-HC 100%	0.001
Ag0-HC 100% vs. Ag180-HC 30%	0.0047
Ag0-HC 100% vs. Ag480-HC 10%	0.008
Ag0-HC 100% vs. Ag180-HC 10%	0.0478
Ag0-HC 100% vs. Ag180-HC 50%	0.0995
Ag0-HC 100% vs. Ag860-HC 30%	0.2512
Ag0-HC 100% vs. Ag2300-HC 10%	0.2897
Ag0-HC 100% vs. Ag180-HC 3%	0.4402
Ag0-HC 100% vs. Ag5200-HC 50%	0.4946
Ag0-HC 100% vs. Ag2300-HC 3%	0.5202

Ag0-HC 100% vs. Ag2300-HC 30%	0.7994
Ag0-HC 100% vs. Ag2300-HC 50%	0.8289
Ag0-HC 100% vs. Ag290-HC 30%	0.9265
Ag0-HC 100% vs. Ag860-HC 10%	0.9805
Ag0-HC 100% vs. Ag290-HC 50%	0.9935
Ag0-HC 100% vs. Ag5200-HC 30%	1
Ag0-HC 100% vs. Ag480-HC 3%	1
Ag0-HC 100% vs. Ag860-HC 50%	1
Ag0-HC 100% vs. Ag0-HC 50%	1
Ag0-HC 50% vs. Ag5200-HC 100%	< .0001
Ag0-HC 50% vs. Ag2300-HC 100%	< .0001
Ag0-HC 50% vs. Ag180-HC 100%	< .0001
Ag0-HC 50% vs. Ag480-HC 100%	< .0001
Ag0-HC 50% vs. Ag480-HC 30%	0.0005
Ag0-HC 50% vs. Ag480-HC 50%	0.0007
Ag0-HC 50% vs. Ag860-HC 100%	0.0012
Ag0-HC 50% vs. Ag290-HC 100%	0.0016
Ag0-HC 50% vs. Ag180-HC 30%	0.007
Ag0-HC 50% vs. Ag480-HC 10%	0.0117
Ag0-HC 50% vs. Ag180-HC 10%	0.0658
Ag0-HC 50% vs. Ag180-HC 50%	0.1322
Ag0-HC 50% vs. Ag860-HC 30%	0.3137
Ag0-HC 50% vs. Ag2300-HC 10%	0.3573
Ag0-HC 50% vs. Ag180-HC 3%	0.5202

Ag0-HC 50% vs. Ag5200-HC 50%	0.5762
Ag0-HC 50% vs. Ag2300-HC 3%	0.6019
Ag0-HC 50% vs. Ag2300-HC 30%	0.8587
Ag0-HC 50% vs. Ag2300-HC 50%	0.8828
Ag0-HC 50% vs. Ag290-HC 30%	0.9563
Ag0-HC 50% vs. Ag860-HC 10%	0.9905
Ag0-HC 50% vs. Ag290-HC 50%	0.9973
Ag0-HC 50% vs. Ag5200-HC 30%	1
Ag0-HC 50% vs. Ag480-HC 3%	1
Ag0-HC 50% vs. Ag860-HC 50%	1
Ag860-HC 50% vs. Ag5200-HC 100%	< .0001
Ag860-HC 50% vs. Ag2300-HC 100%	< .0001
Ag860-HC 50% vs. Ag180-HC 100%	< .0001
Ag860-HC 50% vs. Ag480-HC 100%	< .0001
Ag860-HC 50% vs. Ag480-HC 30%	0.0047
Ag860-HC 50% vs. Ag480-HC 50%	0.0063
Ag860-HC 50% vs. Ag860-HC 100%	0.011
Ag860-HC 50% vs. Ag290-HC 100%	0.0136
Ag860-HC 50% vs. Ag180-HC 30%	0.0486
Ag860-HC 50% vs. Ag480-HC 10%	0.0737
Ag860-HC 50% vs. Ag180-HC 10%	0.2797
Ag860-HC 50% vs. Ag180-HC 50%	0.4484
Ag860-HC 50% vs. Ag860-HC 30%	0.7299
Ag860-HC 50% vs. Ag2300-HC 10%	0.7748

Ag860-HC 50% vs. Ag180-HC 3%	0.895
Ag860-HC 50% vs. Ag5200-HC 50%	0.9228
Ag860-HC 50% vs. Ag2300-HC 3%	0.9337
Ag860-HC 50% vs. Ag2300-HC 30%	0.9938
Ag860-HC 50% vs. Ag2300-HC 50%	0.9959
Ag860-HC 50% vs. Ag290-HC 30%	0.9995
Ag860-HC 50% vs. Ag860-HC 10%	1
Ag860-HC 50% vs. Ag290-HC 50%	1
Ag860-HC 50% vs. Ag5200-HC 30%	1
Ag860-HC 50% vs. Ag480-HC 3%	1
Ag480-HC 3% vs. Ag5200-HC 100%	< .0001
Ag480-HC 3% vs. Ag2300-HC 100%	< .0001
Ag480-HC 3% vs. Ag180-HC 100%	< .0001
Ag480-HC 3% vs. Ag480-HC 100%	< .0001
Ag480-HC 3% vs. Ag480-HC 30%	0.0355
Ag480-HC 3% vs. Ag480-HC 50%	0.0454
Ag480-HC 3% vs. Ag860-HC 100%	0.0714
Ag480-HC 3% vs. Ag290-HC 100%	0.0851
Ag480-HC 3% vs. Ag180-HC 30%	0.2275
Ag480-HC 3% vs. Ag480-HC 10%	0.3067
Ag480-HC 3% vs. Ag180-HC 10%	0.6941
Ag480-HC 3% vs. Ag180-HC 50%	0.853
Ag480-HC 3% vs. Ag860-HC 30%	0.9743
Ag480-HC 3% vs. Ag2300-HC 10%	0.9831

Ag480-HC 3% vs. Ag180-HC 3%	0.9969
Ag480-HC 3% vs. Ag5200-HC 50%	0.9984
Ag480-HC 3% vs. Ag2300-HC 3%	0.9989
Ag480-HC 3% vs. Ag2300-HC 30%	1
Ag480-HC 3% vs. Ag2300-HC 50%	1
Ag480-HC 3% vs. Ag290-HC 30%	1
Ag480-HC 3% vs. Ag860-HC 10%	1
Ag480-HC 3% vs. Ag290-HC 50%	1
Ag480-HC 3% vs. Ag5200-HC 30%	1
Ag5200-HC 30% vs. Ag5200-HC 100%	< .0001
Ag5200-HC 30% vs. Ag2300-HC 100%	< .0001
Ag5200-HC 30% vs. Ag180-HC 100%	< .0001
Ag5200-HC 30% vs. Ag480-HC 100%	< .0001
Ag5200-HC 30% vs. Ag480-HC 30%	0.0446
Ag5200-HC 30% vs. Ag480-HC 50%	0.0566
Ag5200-HC 30% vs. Ag860-HC 100%	0.0879
Ag5200-HC 30% vs. Ag290-HC 100%	0.1042
Ag5200-HC 30% vs. Ag180-HC 30%	0.2668
Ag5200-HC 30% vs. Ag480-HC 10%	0.3535
Ag5200-HC 30% vs. Ag180-HC 10%	0.7452
Ag5200-HC 30% vs. Ag180-HC 50%	0.8878
Ag5200-HC 30% vs. Ag860-HC 30%	0.9837
Ag5200-HC 30% vs. Ag2300-HC 10%	0.9898
Ag5200-HC 30% vs. Ag180-HC 3%	0.9984

Ag5200-HC 30% vs. Ag5200-HC 50%	0.9992
Ag5200-HC 30% vs. Ag2300-HC 3%	0.9995
Ag5200-HC 30% vs. Ag2300-HC 30%	1
Ag5200-HC 30% vs. Ag2300-HC 50%	1
Ag5200-HC 30% vs. Ag290-HC 30%	1
Ag5200-HC 30% vs. Ag860-HC 10%	1
Ag5200-HC 30% vs. Ag290-HC 50%	1
Ag290-HC 50% vs. Ag5200-HC 100%	< .0001
Ag290-HC 50% vs. Ag2300-HC 100%	< .0001
Ag290-HC 50% vs. Ag180-HC 100%	0.0005
Ag290-HC 50% vs. Ag480-HC 100%	0.0007
Ag290-HC 50% vs. Ag480-HC 30%	0.27
Ag290-HC 50% vs. Ag480-HC 50%	0.3172
Ag290-HC 50% vs. Ag860-HC 100%	0.4198
Ag290-HC 50% vs. Ag290-HC 100%	0.4651
Ag290-HC 50% vs. Ag180-HC 30%	0.7528
Ag290-HC 50% vs. Ag480-HC 10%	0.8382
Ag290-HC 50% vs. Ag180-HC 10%	0.9889
Ag290-HC 50% vs. Ag180-HC 50%	0.9987
Ag290-HC 50% vs. Ag860-HC 30%	1
Ag290-HC 50% vs. Ag2300-HC 10%	1
Ag290-HC 50% vs. Ag180-HC 3%	1
Ag290-HC 50% vs. Ag5200-HC 50%	1
Ag290-HC 50% vs. Ag2300-HC 3%	1

Ag290-HC 50% vs. Ag2300-HC 30%	1
Ag290-HC 50% vs. Ag2300-HC 50%	1
Ag290-HC 50% vs. Ag290-HC 30%	1
Ag290-HC 50% vs. Ag860-HC 10%	1
Ag860-HC 10% vs. Ag5200-HC 100%	< .0001
Ag860-HC 10% vs. Ag2300-HC 100%	< .0001
Ag860-HC 10% vs. Ag180-HC 100%	0.0009
Ag860-HC 10% vs. Ag480-HC 100%	0.0013
Ag860-HC 10% vs. Ag480-HC 30%	0.3687
Ag860-HC 10% vs. Ag480-HC 50%	0.4239
Ag860-HC 10% vs. Ag860-HC 100%	0.5374
Ag860-HC 10% vs. Ag290-HC 100%	0.5848
Ag860-HC 10% vs. Ag180-HC 30%	0.8472
Ag860-HC 10% vs. Ag480-HC 10%	0.9106
Ag860-HC 10% vs. Ag180-HC 10%	0.9968
Ag860-HC 10% vs. Ag180-HC 50%	0.9998
Ag860-HC 10% vs. Ag860-HC 30%	1
Ag860-HC 10% vs. Ag2300-HC 10%	1
Ag860-HC 10% vs. Ag180-HC 3%	1
Ag860-HC 10% vs. Ag5200-HC 50%	1
Ag860-HC 10% vs. Ag2300-HC 3%	1
Ag860-HC 10% vs. Ag2300-HC 30%	1
Ag860-HC 10% vs. Ag2300-HC 50%	1
Ag860-HC 10% vs. Ag290-HC 30%	1

Ag290-HC 30% vs. Ag5200-HC 100%	< .0001
Ag290-HC 30% vs. Ag2300-HC 100%	< .0001
Ag290-HC 30% vs. Ag180-HC 100%	0.0024
Ag290-HC 30% vs. Ag480-HC 100%	0.0035
Ag290-HC 30% vs. Ag480-HC 30%	0.5503
Ag290-HC 30% vs. Ag480-HC 50%	0.6105
Ag290-HC 30% vs. Ag860-HC 100%	0.7221
Ag290-HC 30% vs. Ag290-HC 100%	0.7639
Ag290-HC 30% vs. Ag180-HC 30%	0.9451
Ag290-HC 30% vs. Ag480-HC 10%	0.9743
Ag290-HC 30% vs. Ag180-HC 10%	0.9997
Ag290-HC 30% vs. Ag180-HC 50%	1
Ag290-HC 30% vs. Ag860-HC 30%	1
Ag290-HC 30% vs. Ag2300-HC 10%	1
Ag290-HC 30% vs. Ag180-HC 3%	1
Ag290-HC 30% vs. Ag5200-HC 50%	1
Ag290-HC 30% vs. Ag2300-HC 3%	1
Ag290-HC 30% vs. Ag2300-HC 30%	1
Ag290-HC 30% vs. Ag2300-HC 50%	1
Ag2300-HC 50% vs. Ag5200-HC 100%	< .0001
Ag2300-HC 50% vs. Ag2300-HC 100%	0.0002
Ag2300-HC 50% vs. Ag180-HC 100%	0.0056
Ag2300-HC 50% vs. Ag480-HC 100%	0.008
Ag2300-HC 50% vs. Ag480-HC 30%	0.7142

Ag2300-HC 50% vs. Ag480-HC 50%	0.7676
Ag2300-HC 50% vs. Ag860-HC 100%	0.8559
Ag2300-HC 50% vs. Ag290-HC 100%	0.8853
Ag2300-HC 50% vs. Ag180-HC 30%	0.9843
Ag2300-HC 50% vs. Ag480-HC 10%	0.9943
Ag2300-HC 50% vs. Ag180-HC 10%	1
Ag2300-HC 50% vs. Ag180-HC 50%	1
Ag2300-HC 50% vs. Ag860-HC 30%	1
Ag2300-HC 50% vs. Ag2300-HC 10%	1
Ag2300-HC 50% vs. Ag180-HC 3%	1
Ag2300-HC 50% vs. Ag5200-HC 50%	1
Ag2300-HC 50% vs. Ag2300-HC 3%	1
Ag2300-HC 50% vs. Ag2300-HC 30%	1
Ag2300-HC 30% vs. Ag5200-HC 100%	< .0001
Ag2300-HC 30% vs. Ag2300-HC 100%	0.0002
Ag2300-HC 30% vs. Ag180-HC 100%	0.0067
Ag2300-HC 30% vs. Ag480-HC 100%	0.0095
Ag2300-HC 30% vs. Ag480-HC 30%	0.749
Ag2300-HC 30% vs. Ag480-HC 50%	0.7994
Ag2300-HC 30% vs. Ag860-HC 100%	0.8802
Ag2300-HC 30% vs. Ag290-HC 100%	0.9063
Ag2300-HC 30% vs. Ag180-HC 30%	0.9889
Ag2300-HC 30% vs. Ag480-HC 10%	0.9963
Ag2300-HC 30% vs. Ag180-HC 10%	1

Ag2300-HC 30% vs. Ag180-HC 50%	1
Ag2300-HC 30% vs. Ag860-HC 30%	1
Ag2300-HC 30% vs. Ag2300-HC 10%	1
Ag2300-HC 30% vs. Ag180-HC 3%	1
Ag2300-HC 30% vs. Ag5200-HC 50%	1
Ag2300-HC 30% vs. Ag2300-HC 3%	1
Ag2300-HC 3% vs. Ag5200-HC 100%	< .0001
Ag2300-HC 3% vs. Ag2300-HC 100%	0.001
Ag2300-HC 3% vs. Ag180-HC 100%	0.0261
Ag2300-HC 3% vs. Ag480-HC 100%	0.0355
Ag2300-HC 3% vs. Ag480-HC 30%	0.9388
Ag2300-HC 3% vs. Ag480-HC 50%	0.9588
Ag2300-HC 3% vs. Ag860-HC 100%	0.9831
Ag2300-HC 3% vs. Ag290-HC 100%	0.9889
Ag2300-HC 3% vs. Ag180-HC 30%	0.9997
Ag2300-HC 3% vs. Ag480-HC 10%	0.9999
Ag2300-HC 3% vs. Ag180-HC 10%	1
Ag2300-HC 3% vs. Ag180-HC 50%	1
Ag2300-HC 3% vs. Ag860-HC 30%	1
Ag2300-HC 3% vs. Ag2300-HC 10%	1
Ag2300-HC 3% vs. Ag180-HC 3%	1
Ag2300-HC 3% vs. Ag5200-HC 50%	1
Ag5200-HC 50% vs. Ag5200-HC 100%	< .0001
Ag5200-HC 50% vs. Ag2300-HC 100%	0.0012

Ag5200-HC 50% vs. Ag180-HC 100%	0.0291
Ag5200-HC 50% vs. Ag480-HC 100%	0.0394
Ag5200-HC 50% vs. Ag480-HC 30%	0.948
Ag5200-HC 50% vs. Ag480-HC 50%	0.9657
Ag5200-HC 50% vs. Ag860-HC 100%	0.9865
Ag5200-HC 50% vs. Ag290-HC 100%	0.9913
Ag5200-HC 50% vs. Ag180-HC 30%	0.9998
Ag5200-HC 50% vs. Ag480-HC 10%	1
Ag5200-HC 50% vs. Ag180-HC 10%	1
Ag5200-HC 50% vs. Ag180-HC 50%	1
Ag5200-HC 50% vs. Ag860-HC 30%	1
Ag5200-HC 50% vs. Ag2300-HC 10%	1
Ag5200-HC 50% vs. Ag180-HC 3%	1
Ag180-HC 3% vs. Ag5200-HC 100%	< .0001
Ag180-HC 3% vs. Ag2300-HC 100%	0.0016
Ag180-HC 3% vs. Ag180-HC 100%	0.0368
Ag180-HC 3% vs. Ag480-HC 100%	0.0494
Ag180-HC 3% vs. Ag480-HC 30%	0.9646
Ag180-HC 3% vs. Ag480-HC 50%	0.9776
Ag180-HC 3% vs. Ag860-HC 100%	0.992
Ag180-HC 3% vs. Ag290-HC 100%	0.995
Ag180-HC 3% vs. Ag180-HC 30%	0.9999
Ag180-HC 3% vs. Ag480-HC 10%	1
Ag180-HC 3% vs. Ag180-HC 10%	1

Ag180-HC 3% vs. Ag180-HC 50%	1
Ag180-HC 3% vs. Ag860-HC 30%	1
Ag180-HC 3% vs. Ag2300-HC 10%	1
Ag2300-HC 10% vs. Ag5200-HC 100%	< .0001
Ag2300-HC 10% vs. Ag2300-HC 100%	0.0037
Ag2300-HC 10% vs. Ag180-HC 100%	0.0725
Ag2300-HC 10% vs. Ag480-HC 100%	0.095
Ag2300-HC 10% vs. Ag480-HC 30%	0.9916
Ag2300-HC 10% vs. Ag480-HC 50%	0.9955
Ag2300-HC 10% vs. Ag860-HC 100%	0.9989
Ag2300-HC 10% vs. Ag290-HC 100%	0.9994
Ag2300-HC 10% vs. Ag180-HC 30%	1
Ag2300-HC 10% vs. Ag480-HC 10%	1
Ag2300-HC 10% vs. Ag180-HC 10%	1
Ag2300-HC 10% vs. Ag180-HC 50%	1
Ag2300-HC 10% vs. Ag860-HC 30%	1
Ag860-HC 30% vs. Ag5200-HC 100%	< .0001
Ag860-HC 30% vs. Ag2300-HC 100%	0.0048
Ag860-HC 30% vs. Ag180-HC 100%	0.0879
Ag860-HC 30% vs. Ag480-HC 100%	0.114
Ag860-HC 30% vs. Ag480-HC 30%	0.995
Ag860-HC 30% vs. Ag480-HC 50%	0.9975
Ag860-HC 30% vs. Ag860-HC 100%	0.9994
Ag860-HC 30% vs. Ag290-HC 100%	0.9997

Ag860-HC 30% vs. Ag180-HC 30%	1
Ag860-HC 30% vs. Ag480-HC 10%	1
Ag860-HC 30% vs. Ag180-HC 10%	1
Ag860-HC 30% vs. Ag180-HC 50%	1
Ag180-HC 50% vs. Ag5200-HC 100%	< .0001
Ag180-HC 50% vs. Ag2300-HC 100%	0.0186
Ag180-HC 50% vs. Ag180-HC 100%	0.2275
Ag180-HC 50% vs. Ag480-HC 100%	0.2797
Ag180-HC 50% vs. Ag480-HC 30%	0.9999
Ag180-HC 50% vs. Ag480-HC 50%	1
Ag180-HC 50% vs. Ag860-HC 100%	1
Ag180-HC 50% vs. Ag290-HC 100%	1
Ag180-HC 50% vs. Ag180-HC 30%	1
Ag180-HC 50% vs. Ag480-HC 10%	1
Ag180-HC 50% vs. Ag180-HC 10%	1
Ag180-HC 10% vs. Ag5200-HC 100%	< .0001
Ag180-HC 10% vs. Ag2300-HC 100%	0.0423
Ag180-HC 10% vs. Ag180-HC 100%	0.3802
Ag180-HC 10% vs. Ag480-HC 100%	0.4484
Ag180-HC 10% vs. Ag480-HC 30%	1
Ag180-HC 10% vs. Ag480-HC 50%	1
Ag180-HC 10% vs. Ag860-HC 100%	1
Ag180-HC 10% vs. Ag290-HC 100%	1
Ag180-HC 10% vs. Ag180-HC 30%	1

Ag180-HC 10% vs. Ag480-HC 10%	1
Ag480-HC 10% vs. Ag5200-HC 100%	< .0001
Ag480-HC 10% vs. Ag2300-HC 100%	0.1848
Ag480-HC 10% vs. Ag180-HC 100%	0.7712
Ag480-HC 10% vs. Ag480-HC 100%	0.8289
Ag480-HC 10% vs. Ag480-HC 30%	1
Ag480-HC 10% vs. Ag480-HC 50%	1
Ag480-HC 10% vs. Ag860-HC 100%	1
Ag480-HC 10% vs. Ag290-HC 100%	1
Ag480-HC 10% vs. Ag180-HC 30%	1
Ag180-HC 30% vs. Ag5200-HC 100%	< .0001
Ag180-HC 30% vs. Ag2300-HC 100%	0.2543
Ag180-HC 30% vs. Ag180-HC 100%	0.853
Ag180-HC 30% vs. Ag480-HC 100%	0.8973
Ag180-HC 30% vs. Ag480-HC 30%	1
Ag180-HC 30% vs. Ag480-HC 50%	1
Ag180-HC 30% vs. Ag860-HC 100%	1
Ag180-HC 30% vs. Ag290-HC 100%	1
Ag290-HC 100% vs. Ag5200-HC 100%	< .0001
Ag290-HC 100% vs. Ag2300-HC 100%	0.5159
Ag290-HC 100% vs. Ag180-HC 100%	0.976
Ag290-HC 100% vs. Ag480-HC 100%	0.987
Ag290-HC 100% vs. Ag480-HC 30%	1
Ag290-HC 100% vs. Ag480-HC 50%	1

Ag290-HC 100% vs. Ag860-HC 100%	1
Ag860-HC 100% vs. Ag5200-HC 100%	< .0001
Ag860-HC 100% vs. Ag2300-HC 100%	0.5632
Ag860-HC 100% vs. Ag180-HC 100%	0.9837
Ag860-HC 100% vs. Ag480-HC 100%	0.9916
Ag860-HC 100% vs. Ag480-HC 30%	1
Ag860-HC 100% vs. Ag480-HC 50%	1
Ag480-HC 50% vs. Ag5200-HC 100%	< .0001
Ag480-HC 50% vs. Ag2300-HC 100%	0.6778
Ag480-HC 50% vs. Ag180-HC 100%	0.9946
Ag480-HC 50% vs. Ag480-HC 100%	0.9976
Ag480-HC 50% vs. Ag480-HC 30%	1
Ag480-HC 30% vs. Ag5200-HC 100%	< .0001
Ag480-HC 30% vs. Ag2300-HC 100%	0.7337
Ag480-HC 30% vs. Ag180-HC 100%	0.9972
Ag480-HC 30% vs. Ag480-HC 100%	0.9989
Ag480-HC 100% vs. Ag5200-HC 100%	< .0001
Ag480-HC 100% vs. Ag2300-HC 100%	1
Ag480-HC 100% vs. Ag180-HC 100%	1
Ag180-HC 100% vs. Ag5200-HC 100%	< .0001
Ag180-HC 100% vs. Ag2300-HC 100%	1
Ag2300-HC 100% vs. Ag5200-HC 100%	< .0001

Table S5. Results of bacterial reverse mutation test.

Metabolic activation	Dose (%extract/plate)	Number of revertant colonies/plate				
		Base pair substitution mutations			Frameshift mutations	
		TA100	TA1535	WP2*uvrA*	TA98	TA1537
Without	Negative control<br>Physiological saline	140<br>123 (132)	10<br>7 (9)	31<br>22 (27)	22<br>22 (22)	5<br>3 (4)
	1.56	121<br>100 (111)	7<br>8 (8)	30<br>26 (28)	17<br>21 (19)	4<br>3 (4)
	3.13	119<br>121 (120)	6<br>5 (6)	28<br>22 (25)	20<br>19 (20)	2<br>4 (3)
	6.25	145<br>135 (140)	10<br>7 (9)	26<br>24 (25)	20<br>23 (22)	2<br>2 (2)
	12.5	107<br>122 (115)	10<br>12 (11)	22<br>21 (22)	17<br>18 (18)	4<br>2 (3)
	25	127<br>123 (125)	9<br>10 (10)	25<br>30 (28)	16<br>24 (20)	3<br>4 (4)
	50	126<br>141 (134)	7<br>6 (7)	27<br>33 (30)	16<br>18 (17)	5<br>5 (5)
	100	132<br>116 (124)	7<br>11 (9)	25<br>20 (23)	27<br>23 (25)	4<br>5 (5)
With	Negative control<br>Physiological saline	155<br>124 (140)	7<br>12 (10)	40<br>35 (38)	26<br>26 (26)	11<br>11 (11)
	1.56	118<br>141 (130)	11<br>7 (9)	27<br>21 (24)	32<br>28 (30)	7<br>6 (7)
	3.13	154<br>115 (135)	11<br>8 (10)	28<br>20 (24)	31<br>38 (35)	10<br>10 (10)
	6.25	156<br>139 (148)	11<br>13 (12)	26<br>28 (27)	32<br>30 (31)	7<br>6 (7)
	12.5	152<br>128 (140)	9<br>9 (9)	30<br>31 (31)	39<br>34 (37)	8<br>7 (8)
	25	133<br>136 (135)	6<br>7 (7)	32<br>31 (32)	30<br>31 (31)	7<br>10 (9)
	50	154<br>152 (153)	8<br>11 (10)	24<br>34 (29)	30<br>30 (30)	5<br>4 (5)
	100	134<br>127 (131)	12<br>10 (11)	33<br>41 (37)	20<br>27 (24)	3<br>2 (3)
PC Without	Article	4NQO	NaN_3	4NQO	4NQO	9AA
	Dose (μg/plate)	0.1	0.5	0.2	0.2	80
	Colonies/plate	599<br>606 (603)	318<br>344 (331)	244<br>230 (237)	186<br>153 (170)	306<br>263 (285)
PC With	Article	2AA	2AA	2AA	2AA	2AA
	Dose (μg/plate)	1	2	10	0.5	2
	Colonies/plate	659<br>651 (655)	205<br>191 (198)	501<br>474 (488)	290<br>345 (318)	141<br>121 (131)

Remarks
1. ( ): Mean of 2 plates
2. PC: Positive controls
4NQO: 4-Nitroquinoline 1-oxide
NaN_3: Sodium azide
9AA: 9-Aminoacridine hydrochloride monohydrate
2AA: 2-Aminoanthracene

Table S6. General conditions after the administration of test solutions

Test solution	Administration	Animal number	Observation item	Symptom	Time point				
					10 min	4 h	24 h	48 h	72 h
Saline	i.v.	5	Death		0	0	0	0	0
			Appearance	No change	5	5	5	5	5
			Body posture and behavior	No change	5	5	5	5	5
			Nervous system	No change	5	5	5	5	5
			Breathing	No change	5	5	5	5	5
			Hair	No change	5	5	5	5	5
			Oculus	No change	5	5	5	5	5
			Ear, nose, mouth	No change	5	5	5	5	5
			Egestion	No change	5	5	5	5	5
			Other	No	5	5	5	5	5

				change					
Saline extraction liquid of Ag2300-HC	i.v.	5	Death		0	0	0	0	0
			Appearance	No change	5	5	5	5	5
			Body posture and behavior	No change	5	5	5	5	5
			Nervous system	No change	5	5	5	5	5
			Breathing	No change	5	5	5	5	5
			Hair	No change	5	5	5	5	5
			Oculus	No change	5	5	5	5	5
			Ear, nose, mouth	No change	5	5	5	5	5
			Egestion	No change	5	5	5	5	5
			Other	No change	5	5	5	5	5
Saline extraction liquid	i.v.	5	Death		0	0	0	0	0
			Appearance	No change	5	5	5	5	5

of Ag5200-HC			Body posture and behavior	No change	5	5	5	5	5
			Nervous system	No change	5	5	5	5	5
			Breathing	No change	5	5	5	5	5
			Hair	No change	5	5	5	5	5
			Oculus	No change	5	5	5	5	5
			Ear, nose, mouth	No change	5	5	5	5	5
			Egestion	No change	5	5	5	5	5
			Other	No change	5	5	5	5	5
Saline extraction liquid of Ag21000-HC	i.v.	5	Death		0	0	0	0	0
			Appearance	No change	5	5	5	5	5
			Body posture and behavior	No change	5	5	5	5	5
			Nervous	No	5	5	5	5	5

			system	change					
			Breathing	No change	5	5	5	5	5
			Hair	No change	5	5	5	5	5
			Oculus	No change	5	5	5	5	5
			Ear, nose, mouth	No change	5	5	5	5	5
			Egestion	No change	5	5	5	5	5
			Other	No change	5	5	5	5	5
Sesame oil (control)	i.p.	5	Death		0	0	0	0	0
			Appearance	No change	5	5	5	5	5
			Body posture and behavior	No change	5	5	5	5	5
			Nervous system	No change	5	5	5	5	5
			Breathing	No change	5	5	5	5	5
			Hair	No	5	5	5	5	5

				change					
			Oculus	No change	5	5	5	5	5
			Ear, nose, mouth	No change	5	5	5	5	5
			Egestion	No change	5	5	5	5	5
			Other	No change	5	5	5	5	5
Sesame oil extraction liquid of Ag2300-HC	i.p.	5	Death		0	0	0	0	0
			Appearance	No change	5	5	5	5	5
			Body posture and behavior	No change	5	5	5	5	5
			Nervous system	No change	5	5	5	5	5
			Breathing	No change	5	5	5	5	5
			Hair	No change	5	5	5	5	5
			Oculus	No change	5	5	5	5	5
			Ear, nose,	No	5	5	5	5	5

			mouth	change					
			Egestion	No change	5	5	5	5	5
			Other	No change	5	5	5	5	5
Sesame oil extraction liquid of Ag5200-HC	i.p.	5	Death		0	0	0	0	0
			Appearance	No change	5	5	5	5	5
			Body posture and behavior	No change	5	5	5	5	5
			Nervous system	No change	5	5	5	5	5
			Breathing	No change	5	5	5	5	5
			Hair	No change	5	5	5	5	5
			Oculus	No change	5	5	5	5	5
			Ear, nose, mouth	No change	5	5	5	5	5
			Egestion	No change	5	5	5	5	5
			Other	No	5	5	5	5	5

				change					
Sesame oil extraction liquid of Ag21000-HC	i.p.	5	Death		0	0	0	0	0
			Appearance	No change	5	5	5	5	5
			Body posture and behavior	No change	5	5	5	5	5
			Nervous system	No change	5	5	5	5	5
			Breathing	No change	5	5	5	5	5
			Hair	No change	5	5	5	5	5
			Oculus	No change	5	5	5	5	5
			Ear, nose, mouth	No change	5	5	5	5	5
			Egestion	No change	5	5	5	5	5
			Other	No change	5	5	5	5	5

Table S7. Necropsy results after the intravenous administration of test solutions

Organ	Test solution	Saline	Saline extraction liquid of Ag2300-HC	Saline extraction liquid of Ag5200-HC	Saline extraction liquid of Ag21000-HC
	Administration	i.v.	i.v.	i.v.	i.v.
	Animal number	5	5	5	5
Heart	No change	5	5	5	5
Lungs	No change	5	5	5	5
Gastrointestinal tract	No change	5	5	5	5
Liver	No change	5	5	5	5
Spleen	No change	5	5	5	5
Kidneys	No change	5	5	5	5
Genitals	No change	5	5	5	5
Injection part (tail)	No change	5	5	5	5

Table S8. Necropsy results after the intraperitoneal administration of test solutions

Organ	Test solution	Sesame oil	Sesame oil extraction liquid of Ag2300-HC	Sesame oil extraction liquid of Ag5200-HC	Sesame oil extraction liquid of Ag21000-HC
	Administration	i.p.	i.p.	i.p.	i.p.
	Animal number	5	5	5	5
Heart	No change	5	5	5	5
Lungs	No change	5	5	5	5
Gastrointestinal tract	No change	5	5	5	5
Liver	No change	5	5	5	5
Spleen	No change	5	5	5	5
Kidneys	No change	5	5	5	5
Genitals	No change	5	5	5	5
Injection part (abdominal)	No change	5	5	5	5
